# Speed limits: the effects of industrial food processing and food texture on daily energy intake and eating behaviour in healthy adults

**DOI:** 10.1007/s00394-023-03202-z

**Published:** 2023-07-14

**Authors:** Marlou Lasschuijt, Guido Camps, Monica Mars, Els Siebelink, Kees de Graaf, Dieuwerke Bolhuis

**Affiliations:** 1 Division of Human Nutrition and Health, Wageningen University & Research, Stippeneng 4, 6708 WE Wageningen, The Netherlands; 2 Food Quality and Design Group, Wageningen University & Research, Bornse Weilanden 9, 6708 WG Wageningen, The Netherlands

**Keywords:** NOVA, Energy intake rate, Food intake, Industrial processing, Diet, Food texture

## Abstract

**Purpose:**

Frequent consumption of industrially processed foods has been associated with obesity. However, it is unknown what drives this association. Food textures of industrially processed foods that stimulate energy overconsumption may be an important driver of this association. Therefore, this study aimed to determine the independent and combined effects of food texture and level of industrial food processing (based on the NOVA classification) on daily energy intake and eating behaviour.

**Methods:**

Eighteen healthy adults (F/M: 11/7, 23 ± 3 y, 22.1 ± 2.0 kg/m^2^) participated in a 2 × 2 randomized crossover dietary intervention with four conditions (total of 288 meals): hard unprocessed, hard (ultra-)processed, soft unprocessed and soft (ultra-)processed. Daily diets were offered ad libitum and were equal in energy density (1 kcal/g). Food Intake (g) was measured by pre- and post-consumption weighing of the plates. Eating behaviour parameters were derived from video annotations.

**Results:**

Daily energy intake and food intake were, respectively, 33% (571 ± 135 kcal) and 14% (247 ± 146 g) lower in the hard compared to the soft conditions (main texture *p* < 0.001). Energy intake was lower in both hard conditions compared to the (ultra)processed soft condition (Tukey *p* < 0.04). Eating rate (g/min) was on average 85% slower (*P* < 0.001) in the hard compared to the soft conditions (*p* < 0.001). Level of processing did not affect food intake.

**Conclusion:**

Consumption of hard-textured foods reduces daily energy intake of (ultra-) processed foods. This preliminary investigation shows that there is great variability in food properties that affect energy and food intake beyond industrial food processing. However, findings should be interpreted with precaution considering the limited sample size of this trial. Future classification systems for public health messaging should include energy intake rate to help reduce overconsumption.

**Clinical trial registry:**

NCT04280146, https://www.clinicaltrials.gov, February 21st 2020.

**Supplementary Information:**

The online version contains supplementary material available at 10.1007/s00394-023-03202-z.

## Introduction

The number of people with overweight or obesity has increased to pandemic proportions during the last century [1]. This increase has coincided with the upscaling of food production through food processing as part of the industrial food revolution [2, 3]. Food processing includes any transformation from raw agricultural products to edible food products. Simple to highly advanced techniques are used to process foods, ranging from rinsing, cutting or heating on household levels to more complex industrial processing, such as extracting, extruding, fermenting, pressuring and hydrogenating. Historically, the primary aim of industrial food processing is to produce accessible, safe, yet palatable foods with extended shelf lives, whereas more recently the emphasis has shifted towards the production of sustainable and healthy food products [4, 5].

The level of food processing can be, amongst others, categorized based on the NOVA classification [6]. This classification consists of four categories: (1) unprocessed to minimally processed foods, (2) processed culinary ingredients, (3) processed foods including canned or bottled vegetables, fruits in syrup and salted nuts and (4) ultra-processed foods (UPF), which are defined as: “Formulations of ingredients, mostly of exclusive industrial use, that result from a series of industrial processes” [6].

This NOVA classification system is often used in nutritional epidemiological studies to assess associations between dietary intake of ultra-processed foods with health-related outcomes. A recent meta-analysis of studies (*n* = 14) investigating the association between weight status and dietary intake of ultra-processed foods concluded that diets high in industrial processed foods are positively associated with BMI [7]. To date, the findings of these observational studies have been confirmed by one experimental study. In that crossover inpatient study, 20 participants received a 2-week diet that was either high in ultra-processed or unprocessed foods and matched for energy content and palatability [8]. Participants on the ultra-processed diet showed higher energy intakes (500 kcal/day) and gained weight (0.9 kg/2 weeks) compared to a diet of low-processed foods [8]. However, the underlying (biological) mechanism remains unknown [9].

Food properties that are hypothesized to drive or moderate the association between ultra-processed foods and weight gain are high energy density, low micronutrient density, food texture properties that lead to a fast eating rate, and hyper palatability due to high salt, sugar and fat content [8, 10–12].

For factors such as energy density and food texture, there is ample experimental evidence that they affect food and energy intake [13–16]. Energy intake is affected by the energy density of the food consumed. Individuals tend to consume a consistent weight of food when energy density, but not other meal or food product properties, are varied. [17]. Contrary to energy density, food texture does affect the amount of food consumed. Food texture affects intake through eating behaviour parameters that determine oro-sensory exposure duration (oral residence time) and eating rate [13, 14, 18]. For example, hard, compared to soft, food textures lead to a slower eating rate due to longer oral processing time per bite or gram of food (chewing duration). Together these oral processing components onset satiation. Because of this, hard-textured, slowly consumed foods are eaten in smaller amounts compared to soft-textured, fast consumed foods [19]. Food texture combined with the energy density of a food determines the energy intake rate that is also known to play an important role in food and energy intake [20]. However, energy density and texture food properties vary greatly among foods within each NOVA category and therefore the extend in which they drive or moderate the association between excess calorie intake and industrial food processing is unknown [15]. The objective of the current study was to determine the independent and additive effects of industrial food processing and food texture on daily energy intake and eating behaviour. We hypothesized that a slower eating rate, induced by texture manipulations (hardness), would decrease energy intake over the course of the day, independent of industrial processing level.

## Methods and materials

### Study design

A 2 × 2 crossover study was set up with one-day diets of two levels of texture and two levels of industrial processing: hard unprocessed, hard (ultra-)processed, soft unprocessed and soft (ultra-)processed foods. Food texture was manipulated to create diets that consisted of hard-, and soft-, textured foods such that meals were consumed with relatively slow and fast eating rates. Industrial processing level of the diets was based on the NOVA classification, Food and Agriculture Organization of the United Nations (FAO) definitions by Monteiro et al. [6]. Participants were exposed to each of the four 1-day diets in randomised order, and test days were separated by approximately a week. On test days participants consumed three meals (breakfast, lunch and dinner + dessert) at the eating behaviour research unit of Wageningen University and were provided with two take-home snacks.

#### Participant recruitment and characteristics

The study was performed between October and December 2020 at the Human Nutrition Research Unit of Wageningen University and Research, The Netherlands. Healthy adult participants (18–55 years, BMI: 18.5–30 kg/m^2^) were recruited from the volunteer database of the division, and additional advertisements were posted on social media. Potential participants were informed about the study during an online information meeting, after which they were asked to sign informed consent and completed a questionnaire to assess eligibility criteria. Participants were not informed about the true aim but were told that the study investigated cultural differences in eating behaviour. When the study was finished, participants were debriefed and informed about the true aim. Only two participants guessed the aim of the study correctly.

Inclusion criteria were: being able to understand and speak English, (commonly) eat three meals a day around the same time, weekends not included. Exclusion criteria were: food allergies for any of the foods offered during the study, a lack of appetite or any dental chewing or swallowing problems, following an energy-restricted diet, gained or lost > 5 kg of body weight during the past two months, drinking more than 21 glasses of alcohol per week, using medication that influencing appetite or taste or smell, performing intensive exercise for more than 8 h a week, or being a high restrained eater (i.e. chronic tendency to limit food intake to lose weight or to prevent weight gain) according to the Dutch Eating Behaviour Questionnaire (DEBQ): cut-off scores > 2.89 for men and > 3.39 for women [21].

During the screening part of the information meeting, participants rated liking of all foods items that would be offered during the study on a 9-point Likert scale (based on descriptions of the food items and meals) or could indicate that they were unfamiliar with the food items. Participants were not eligible if they disliked (score < 5) or were unfamiliar with one or more food items that would be offered during the main meals. Personnel and Master thesis students of the Division of Human Nutrition and Health were not allowed to participate. After screening, eligible participants were invited for height and body weight measures. Based on these measurements, participants were excluded if their BMI was outside the 18.5–30 kg/m^2^ range.

**Fig. 1:**
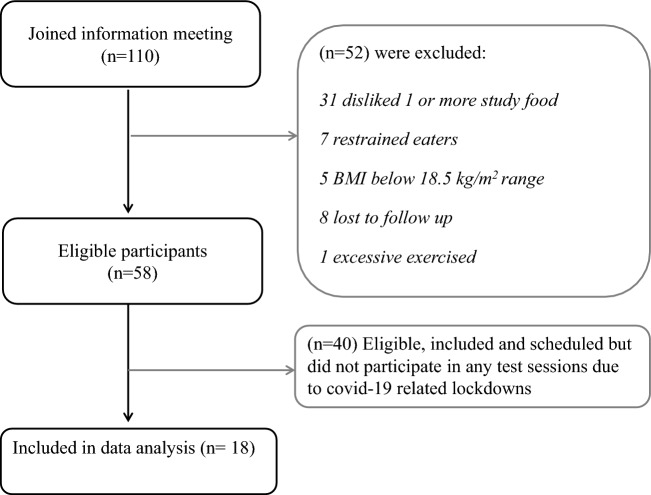
Flowchart of included and excluded participants of the study. Based on participant availability and scheduling, the first 18 were included in the data analysis reported in this paper. The study was early terminated due to COVID-19

In total, 110 participants were invited for the information meeting, of which 58 were eligible to participate in the study. Finally, 18 (7 males) were included and completed the study and were included in the data analysis (Fig. 1). Due to COVID-19 restrictions, the study was terminated early, see sample size paragraph.

The study was approved by the Social Ethical Committee of Wageningen University, The Netherlands (Lasschuijt, 2020–2011). Participants received financial compensation for their time and effort.

#### Sample size

Based on previous studies, the estimated sample size as reported in the pre-registration was *n* = 60, to show a 10% difference in intake between conditions (α = 0.05, power 1 − β = 0.80). This was based on previous studies with a similar study design by Bolhuis et al. [22, 23]. Due to COVID-19 and associated lockdowns in December 2020–March 2021, an unplanned interim data analysis was done to determine a better sample size estimation based on variation in the data collected till date (*n* = 18). At interim the effect size was larger than expected (14%, instead of 10%) which compensated for the higher variance in the data due to the smaller sample size. Due to the continuity of the lockdown, it was therefore decided to prematurely terminate the study and include 18 participants in the data analysis. Due to this early closure of participant inclusion, this trial may be underpowered to find smaller (10% instead of 14%) differences between study conditions. Because of these unforeseen circumstances, we now refer to the study as a pilot trial.

### Study procedures

**Fig. 2:**
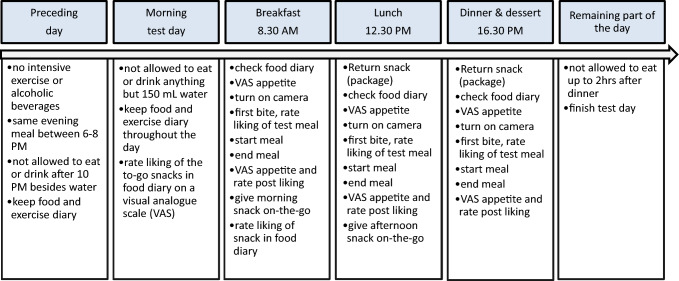
Overview of study day procedures. Meals were according to one of the four study conditions, unprocessed hard, unprocessed soft, (ultra-)processed hard and (ultra-)processed soft

For an overview of the study procedures, see Fig. 2. Participants were given instructions not to drink any alcoholic drinks or do intensive exercise 24 h before each test day, to eat the same meal (of their own choice) between 6 and 8 PM and not to eat or drink anything after 10 PM besides water the evening preceding each test day. On the morning of the test day, participants were not allowed to eat or drink anything besides a small glass of water (150 mL). On test days participants came to the eating behaviour research unit (diner room) to eat their main meals (breakfast, lunch, dinner and desserts). Participants ate alone at a Table 2 metres apart and separated by screens. After the breakfast and lunch meal, participants left the research unit and were given a snack (fixed portion).When participants returned for the following meal, intake of the snack was measured by weighing back the left overs and package. During the entire test day, participants were only allowed to eat the food provided by the researchers. Participants were not allowed to share their take-home snacks with anyone. In between main meals participants were only allowed to drink water, tea and coffee without sugar or milk. Participants were asked to keep a food diary the day before and the day of the test sessions. At the end of the test day, participants were instructed not to eat or drink anything up to two hours after finishing their dessert.

Test days were scheduled on the same weekday, one week apart. To accommodate two groups for each main meal, two timeslots were available, and participants always participated in the same round. In round 1, breakfast was served at 8.30 AM, lunch at 12.30 PM and dinner at 4.30 PM, and in round 2 breakfast was served at 9.30 AM, lunch 1.30 PM and dinner 5.30 PM. Due to coronavirus-related rescheduling, some participants (*n* = 6) had their last two sessions in the same week, at least one day apart, and these participants were split into two smaller groups per session and therefore their meals may have started half an hour earlier or later than the original schedule. Number of days between sessions was not a significant covariate to the main outcome model (*p* = 0.56).

Upon arrival, participants’ food diary was checked for compliance and participants were asked to fill in an appetite questionnaire (ratings on a visual analogue scale). They would then receive their meal to taste and rate the palatability of the meal (after the first bite). Participants were instructed to eat as much or as little as they wanted until they felt comfortably full and were recorded on video to assess eating behaviour post-hoc. Participants were asked to raise their hand to indicate when they were done eating and were instructed to stay for 45 min. to consume their meal and they were not allowed to leave earlier. Two participants finished lunch and dinner meals on two occasions and were given an additional plate every time upon finishing the first.

### Intervention diets

Industrial processing level of the diets was based on the NOVA classification [6]; diets were either predominantly from category 1 (unprocessed, 81–84% of provided energy) or from category 3 and 4 (processed and ultra-processed, 72–81% of provided energy). Table 1 provides an overview of the energy provided by the snacks and meals per NOVA class. A detailed overview of all meals, their ingredients and their NOVA category is presented in Supplement 1.

**Table 1:** Amount of daily energy (%) offered from the four NOVA categories for all four study conditions

NOVA category		Study condition: daily diet			
		Unprocessed		(Ultra-)processed	
		Hard	Soft	Hard	Soft
Group 1	Unprocessed or minimally processed foods	84	81	27	19
Group 2	Processed culinary ingredients	16	19	1	0
Group 3	Processed foods	0	0	43	29
Group 4	Ultra-processed foods	0	0	29	52

Food texture was manipulated to create diets that consisted of hard- and soft-textured foods. Compared to soft-textured foods, hard textures were expected to be consumed relatively slow, with small bites, many chews and thus long oro-sensory exposure duration [20]. Food texture manipulations included structural differences such as liquids, semi-solids and solids, and manipulations within a texture category such as variations in hardness and size of vegetable pieces or through use of products that were naturally more elastic or chewy (see Table 2 for an overview of the texture manipulations).

**Table 2:** Overview of the daily diets for each of the four study conditions, including the type of texture manipulation

Meal	Unprocessed		(Ultra-)processed		Type of texture manipulation
	**Hard**	**Soft**	**Hard**	**Soft**	
Breakfast	Fresh mixed fruit	Homemade smoothie	Canned mixed fruit	Store bought smoothie	Solid/liquid
Morning snack	Apple pieces	Apple sauce -no additives	Apple pieces	Store bought Apple juice	Solid/semi-solid/liquid
Lunch	Fresh Tagliatelle pasta with homemade tomato sauce, hard-steamed vegetables and large pieces of chicken fillet	Fresh tagliatelle pasta with homemade tomato sauce, soft-steamed vegetables and homemade pulled chicken	Store bought, pork meat tortellini with pre-canned tomato sauce, hard-cooked vegetables and grated cheese	Ready-to-eat macaroni Bolognese with grated cheese	Hardness and piece size
Afternoon snack	Dried mango	Raisins	Muesli bar pieces	Fruit bar pieces	Elasticity and hardness
Dinner	Potato parts with large pieces of pork fillet and whole hard-steamed green beans	Homemade mashed potato with eggs small pieces of soft-steamed green beans	Pre-flavoured and baked potato parts with large pieces of chicken schnitzel and whole, hard-steamed green beans	Mashed potato with chicken meatballs and small pieces of soft-steamed green beans	Hardness piece size
Dessert	Dried figs and almonds	Curd with added honey and crushed pecan nuts	Mass produced fig bread	Walnut and honey flavoured yoghurt	Solid versus semi-solid

The energy and nutrient composition of the meals and diets were calculated based on the current Dutch food composition table (NEVO table 2019, version 6) and package information by the use of nutrition calculation software (Compleat© 2010–2022 Human Nutrition, Wageningen University). Main meals were served in surplus (ad libitum), that is, 200–300% of a regular portion size. The offered diets were designed to contain similar amounts of energy (between 4435 and 4506 kcal per day) and energy density (all diets; ~ 1 kcal/g), macronutrient composition, as well as on portion size (weight) (Table 3, Supplement 2 for an overview of the meals). All recipes were extensively piloted by research dieticians so that the ingredients and preparation procedures were all standardized before conducting the study.

**Table 3:** Weight and nutritional composition of the daily diets (as served) for each of the four study conditions (Nutritional composition per meal and snack can be found in Supplement 2)

	Unprocessed		(Ultra-)processed	
	Hard	Soft	Hard	Soft
Weight, g	4276	4286	4440	4315
Energy, MJ (kcal)	18.7 (4437)	18.7 (4467)	18.9 (4505)	18.5 (4435)
Energy density, kcal/g	1.0	1.0	1.0	1.0
Fat, gram (en%)	120 (24)	204(40)	136 (27)	216 (43)
Saturated fat, g	22	46	51	74
Total carbohydrate (g(en%))	605 (55)	467(42)	602 (54)	463 (42)
Mono- and disaccharides, g	450	323	336	238
Protein, g (en%)	185 (17)	153 (14)	149 (13)	131 (12)
Fibre, g	96	68	110	43
Sodium, mg	440	640	6184	4681

Note: all main meals and desserts were offered in surplus (ad libitum), which meant 200–300% of the normal portion size

All meals were served with access to a can with 1-L tap water. As the sodium content of the (ultra-)processed meals was higher compared to the non-processed meals (Table 3, overview of meal in Supplement 2), salt and pepper sachets were placed on the table during each meal for participants to add.

### Study measures

#### Food intake

All plates, bowls, packages, sachets and cans were covertly weighed before and after the meals to determine food, water and salt intake. As dinner consisted of three separate (non-mixed) food components (potatoes, beans and egg/meat) on one plate, all components were weighed individually. All food items and ingredients were weighed on a digital scale with an accuracy of 1 g. Energy and macronutrient and salt intake were then calculated by the use of package information and the Dutch Food Composition Database (NEVO table 2019, version 6).

#### Eating behaviours

Participants were video-recorded during the main meals to annotate eating behaviours during the meal. In order to support the video annotations, participants were asked to hold up a numbered card to indicate that they were beginning their meal and raise their hand to indicate when they were finished with eating. For the recordings a webcam was positioned in front of the participant (face-on) at approximately 1.5 m distance where the lower frame was in line with the table, and the upper frame above the top of the cranium and the sides at shoulder width. Video recordings were annotated with the use of Noldus Observer XT 11 (Noldus Information Technology, the Netherlands). Behaviours that were noted were meal duration (min), oro-sensory exposure duration (s), duration between bites (s), number of chews and number of bites during the meal. From these variables, bite size was calculated by the total amount eaten (g) divided by the total number of bites during the meal. Eating rate (g/min) was calculated amount eaten (g) divided by meal duration (min), and energy intake rate was calculated by the energy eaten (kcal) divided by meal duration (min). Due to technical errors, 18 videos are missing and eating behaviour could not be annotated.

#### Appetite and palatability ratings

Before and directly after consumption of meals, participants rated their hunger, fullness, thirst and desire to eat on a 100 mm visual analogue scale (VAS) anchored at 0 with ‘not at all’ and at 100 with ‘extremely’. Additionally, participants rated the palatability of the test meals before and after consumption on a 9-point Likert scale.

### Statistical analyses

Statistical analyses were performed using SAS (version 9.4; SAS Institute Inc., Cary, NC, USA). Data are presented as means ± SEM, unless otherwise stated. *p* values < 0.05 were considered statistically significant.

Mixed-model ANOVA (PROC MIXED) with covariance structure compound symmetry (CS) was used to test effects of fixed factors (processing condition, eating rate condition) and their interaction, on the primary outcomes: food intake (g) and energy intake (kcal) and the other study outcomes: eating behaviour, appetite ratings at baseline and changes in appetite, palatability ratings and sodium intake. Participant number was added as random variable to the models. If main effects or interactions were statistically significant, Tukey’s HSD was used to compare means between study conditions. Before statistical analyses, all outcomes were inspected visually for normality (histogram, Q–Q plot) and tested for order effects. The variables energy intake (kcal) and food intake (g), ratings of thirst, desire to eat, fullness and eating rate (g/min) were not normally distributed; therefore, these variables were log-transformed (using the LOG() function) before adding them to the mixed model. For these variables, geometric means and back-transformed SEs were reported [24]. For total daily meal duration and duration of the dinner, an order effect was found. To correct for these effects, the order of the conditions was added as covariate to the models. Pearson correlations were used to exposure associations between outcome variables.

## Results

### Participant characteristics

Participants (*n* = 18) were on average 23 ± 3 years old and had a BMI of 22.1 ± 2.0 kg/m^2^ (range: 20–26 kg/m^2^) and an average DEBQ restraint score of 2.3 ± 0.3 (men) and 2.5 ± 0.6 (women).

### Main outcome

**Fig. 3:**
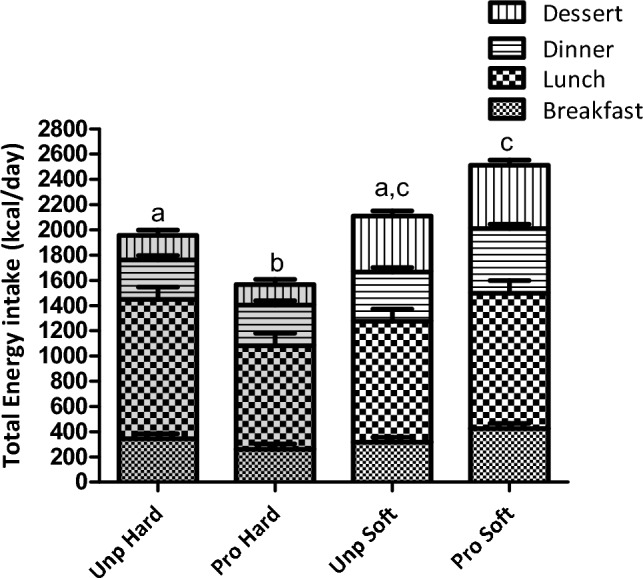
Daily energy intake (kcal) across main meals (excluding snacks) per study condition: unprocessed hard, unprocessed soft, (ultra-)processed hard and (ultra-)processed soft (mean ± SE)

**Fig. 4:**
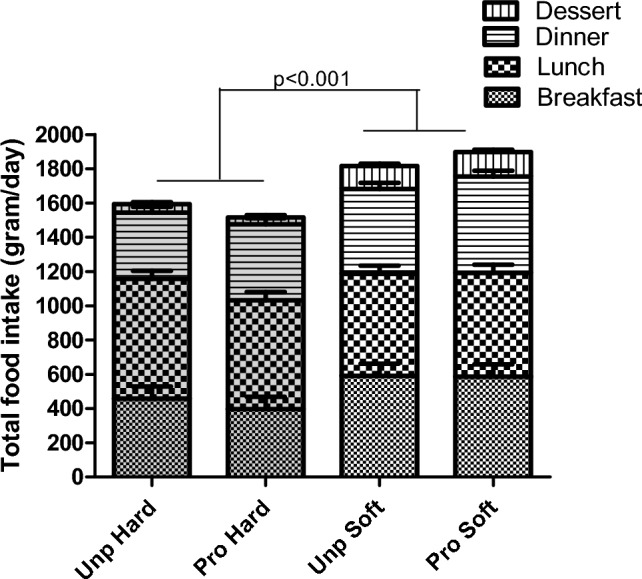
Daily food intake (gram) across main meals (excl. snacks) per study condition; unprocessed hard, unprocessed soft, (ultra-)processed hard and (ultra-)processed soft (mean ± SE)

Overall, daily energy intake was 571 ± 135 kcal lower (33%) in the hard compared to the soft texture condition (main effect texture: *p* < 0.001) (Fig. 3**)**. A significant interaction effect was found where energy intake was lower in both hard texture conditions compared to the (ultra)processed soft texture condition (processing × texture: *p* < 0.001; Tukey *p* < 0.04) (Table 4). In terms of food intake, participants ate 247 ± 146 g (14%) less food in the hard texture conditions compared to the soft texture condition (main effect texture: *p* < 0.001) (Fig. 4 and Table 4). Daily food intake was similar between the unprocessed and (ultra)processed conditions, that is 1737 ± 146 g and 1762 ± 146 g, respectively (main effect processing: *p* = 0.72).

**Table 4:** Energy intake, food intake, eating behaviour and sodium consumption for the four different study conditions (mean ± SE, *n* = 18)

	Unprocessed		(Ultra-)processed		ANOVA fixed effect *p* value		
	Hard	Soft	Hard	Soft	Processing	Texture	Processing × texture
Energy intake (kcal) excl. snacks*	1892 ± 135^a^	2078 ± 137^a,c^	1501 ± 135^b^	2450 ± 133^c^	0.42	< 0.001	< 0.001
Energy intake (kcal) incl. snacks*	1999 ± 134^a,b^	2205 ± 137^b^	1706 ± 139^a^	2608 ± 136^c^	0.91	< 0.001	0.002
Food intake (g) excl. snacks*	1574 ± 145	1736 ± 146	1485 ± 146	1816 ± 146	0.84	< 0.001	0.15
Food intake (g) incl. snacks*	1644 ± 145	1830 ± 146	1609 ± 146	1916 ± 146	0.72	< 0.001	0.34
Energy intake rate ~ ~ (kcal/min)	38 ± 5	62 ± 5	38 ± 5	77 ± 5	0.04	< 0.001	0.11
Eating rate (g/min) ~ ~	30 ± 5	52 ± 5	31 ± 5	61 ± 5	0.12	< 0.001	0.20
Total meal duration ~ ~ (min)	13 ± 1	11 ± 1	12 ± 1	10 ± 1	< 0.001	< 0.001	0.57
OSE duration ~ ~ (s/bite)	18 ± 1	11 ± 1	21 ± 1	10 ± 1	0.34	< 0.001	0.06
Chews per bite ~ ~	20 ± 2	9 ± 2	21 ± 2	8 ± 2	0.74	< 0.001	0.48
Bite size ~ ~ (g/bite)	11 ± 1	16 ± 1	12 ± 1	18 ± 1	0.16	< 0.001	0.42
Sodium in meal (mg)	181 ± 129	206 ± 129	2485 ± 129	2573 ± 132	< 0.001	0.60	0.77
Sodium added (mg)	293 ± 70	437 ± 70	105 ± 70	204 ± 71	< 0.001	0.02	0.63
Sodium total intake (mg)	474 ± 173	643 ± 173	2591 ± 173	2642 ± 173 ~	< 0.001	0.36	0.63

Means with different superscript letters are significantly different. OSE = oro-sensory exposure

*Geometric mean, SE of the original distribution mixed

~ Added salt was not recorded for two subjects during the soft ultra-processed meal

~  ~ 18 observations are missing due to missing video recording

To account for meal fibre and protein content differences, fibre and protein content were added as a covariate to the main model, but these factors did not explain significant variance. Breakfast fibre content was a significant covariate (*p* = 0.02), explaining a proportion of variance in intake of energy during lunch, however main effects were unchanged. Main outcome analyses were run including and excluding outliers, but the outliers did not change the significance of main effects or post-hoc mean difference tests; all results reported are based on the total dataset (*n* = 18).

### Eating behaviour characteristics

**Fig. 5:**
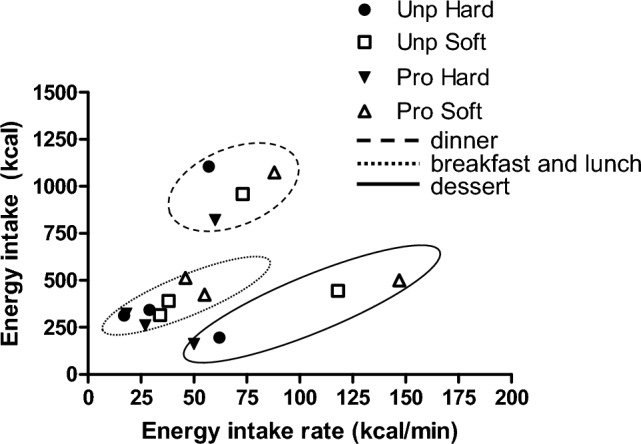
Energy intake vs. energy intake rate per study condition for each main meal and dessert

**Fig. 6:**
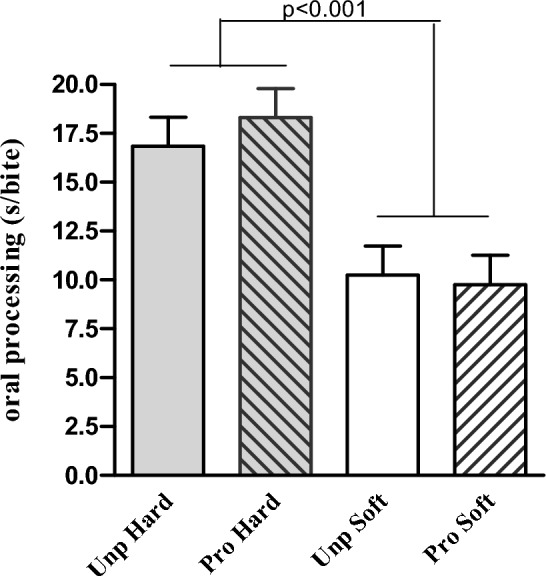
Oro-sensory exposure duration (sec/bite) per study condition; unprocessed hard, unprocessed soft, (ultra-)processed hard and (ultra-)processed soft (mean ± SE)

All eating behaviour characteristics were affected by the food texture manipulations. Energy intake rate (kcal/min) was 82% (38 ± 5 kcal/min) higher in the soft compared to the hard texture conditions (main effect texture *p* < 0.001) and higher in the ultra-processed soft condition compared to the unprocessed soft condition (main effect processing *p* = 0.04) (Table 4**)**. Across all conditions, energy intake rate was positively correlated with energy intake (*r* = 0.72, *p* < 0.001) (Fig. 5**)**. Eating rate (g/min) was on average 46% (26 ± 5 g/min) higher in the soft compared to the hard conditions (*p* < 0.001). Oro-sensory exposure time was twice as long in the hard compared to the soft conditions (*p* < 0.001), and participants chewed twice as many times on each bite of food in the hard compared to the soft conditions (20 ± 2 vs 9 ± 2, *p* < 0.001) (see Fig. 6). Average bite size was 17 ± 1 g in the soft and 12 ± 1 g in the hard texture conditions (*p* < 0.001). Finally, the meal duration was on average 2 ± 0.4 min longer in the hard compared to the soft conditions (*p* < 0.001) and 1 ± 0.3 min longer in the unprocessed compared to processed conditions (*p* = 0.007) (Supplement 3, overview of food and energy intake and eating behaviour characteristics per study meal). Texture and industrial processing level significantly affected meal duration. Participants ate slightly longer in the unprocessed compared to the ultra-processed meals and longer for the hard- vs soft-textured meals. People extended their meal duration with 2–3 min in the unprocessed hard texture condition compared to the other conditions; however, this was not statistically significantly (*p* > 0.05). Due to the longer oral processing time per bite, the extended meal duration did not result in additional food intake.

### Palatability and appetite ratings

The palatability ratings of the meals were all between 6.1 and 7.9 on a 9-point scale. Palatability ratings of the unprocessed breakfast were rated on average one point higher compared to the (ultra-)processed conditions (main effect processing *p *= 0.03). Average palatability of the lunch meals was rated 7.1 ± 0.3, with no differences between study conditions. For the dinner meal, the potato and protein source were liked better in the (ultra)processed conditions compared to the unprocessed conditions (main effect processing: *p* < 0.003). Desserts were rated similar in palatability for all study conditions (Table 5, overview of palatability and appetite ratings.

**Table 5:** Appetite and liking ratings (100 mm visual analogue scale) per meal and study condition

	Unprocessed		(Ultra)-processed		ANOVA fixed effect *p *value		
	Hard	Soft	Hard	Soft	Processing	Texture	Processing* texture
Breakfast							
Hunger							
Pre	64 ± 5	68 ± 5	62 ± 55	69 ± 5	0.81	0.21	0.78
Post	13 ± 4	14 ± 4	18 ± 4	12 ± 4	0.52	0.28	0.14
Fullness							
Pre	10 ± 4	8 ± 4	11 ± 4	8 ± 4	0.89	0.08	0.84
Post	72 ± 4	68 ± 4	58 ± 4	74 ± 4	0.31	0.2	0.05
Thirst							
Pre	71 ± 4	72 ± 4	67 ± 4	67 ± 4	0.14	1.86	0.93
Post	18 ± 4	26 ± 4	24 ± 4	17 ± 4	0.53	0.76	0.01
Desire to eat							
Pre	74 ± 4	69 ± 4	69 ± 4	71 ± 4	0.61	0.62	0.22
Post	16 ± 5	13 ± 5	23 ± 5	18 ± 6	0.1	0.26	0.94
Palatability							
Pre	7.9 ± 0.3^a^	6.8 ± 0.3^b^	6.4 ± 0.3	7.0 ± 0.3	0.03	0.3	0.003
Lunch							
Hunger							
Pre	81 ± 3	74 ± 3	73 ± 3	75 ± 3	0.17	32	0.07
Post	4 ± 3	8 ± 3	6 ± 3	5 ± 3	0.64	0.37	0.11
Fullness							
Pre	10 ± 4	11 ± 4	12 ± 4	12 ± 4	0.89	0.08	0.84
Post	85 ± 4	66 ± 4	78 ± 4	84 ± 4	0.4	0.37	0.12
Thirst							
Pre	49 ± 5	56 ± 5	50 ± 5	53 ± 5	0.78	0.28	0.62
Post	19 ± 5	21 ± 5	33 ± 5	24 ± 5	0.04	0.32	0.13
Desire to eat							
Pre	80 ± 4	74 ± 4	75 ± 4	74 ± 4	0.39	0.25	0.4
Post	9 ± 3	9 ± 3	4 ± 3	6 ± 3	0.04	0.42	0.54
Palatability							
Pre	7.1 ± 0.3	6.6 ± 0.3	7.3 ± 0.3	7.2 ± 0.3	0.11	0.26	0.5
Dinner							
Hunger							
Pre	54 ± 5	47 ± 5	50 ± 5	50 ± 5	0.95	0.33	0.35
Post	10 ± 4	12 ± 4	6 ± 4	7 ± 4	0.004	0.27	0.82
Fullness							
Pre	34 ± 5	33 ± 5	36 ± 5	32 ± 5	0.65	0.82	0.86
Post	78 ± 3	72 ± 3	83 ± 3	85 ± 3	0.03	0.57	0.29
Thirst							
Pre	55 ± 5	57 ± 5	55 ± 5	54 ± 5	0.68	0.89	0.65
Post	26 ± 5	25 ± 5	27 ± 5	24 ± 5	0.95	0.49	0.73
Desire to eat							
Pre	49 ± 4	53 ± 4	53 ± 4	54 ± 4	0.16	0.77	0.44
Post	12 ± 4	14 ± 4	6 ± 4	9 ± 4	0.03	0.33	0.62
Palatability Pre							
Beans	6.7 ± 0.4	6.6 ± 0.4	7.0 ± 0.4	6.4 ± 0.4	0.79	0.13	0.2
Potatoes	6.4 ± 0.4	6.6 ± 0.4	7.4 ± 0.4	7.3 ± 0.4	0.003	0.86	0.52
Protein (meat/egg)	6.5 ± 0.4	6.6 ± 0.4	7.9 ± 0.4	7.7 ± 0.4	< 0.001	0.8	0.67
Dessert (directly after dinner)							
Hunger							
Post	6 ± 3	4 ± 3	4 ± 3	3 ± 3	0.16	0.29	0.72
Fullness							
Post	84 ± 3	87 ± 3	80 ± 3	90 ± 3	0.99	0.16	0.44
Thirst							
Post	29 ± 6	23 ± 6	25 ± 6	22 ± 6	0.58	0.26	0.65
Desire to eat							
Post	8 ± 3	3 ± 3	5 ± 3	4 ± 3	0.43	0.04	0.24
Palatability							
Pre	6.3 ± 0.4	6.1 ± 0.4	6.2 ± 0.4	6.3 ± 0.4	0.86	0.73	0.66

All appetite ratings before the meals were similar between study conditions (all *p* values > 0.05, see Table 5). Appetite ratings after the meals did not differ between study conditions after breakfast, lunch and dessert. However, after dinner, hunger was somewhat higher (4–6 mm on 100 mm VAS) after the unprocessed conditions compared to the (ultra) processed conditions, in line with higher desire to eat and lower fullness ratings. After the dessert meal, no differences in appetite ratings were found between study conditions.

## Discussion

The results of this preliminary investigation show that harder food textures reduce daily energy and food intake of (ultra-) processed foods in a controlled laboratory setting. Food texture affected all eating behaviour characteristics; harder meals were consumed at a slower eating rate, with twice as many chews and longer oro-sensory exposure times. Daily energy and food intake were, respectively, 33% and 14% lower in the hard compared to the soft texture conditions.

That food texture affects eating behaviour, and food intake has repeatedly been shown by single-meal studies [18, 23, 25–30]. However, this study is among the first to show that food texture manipulations can affect daily energy and food intake in a full day semi-controlled experimental trial. Our findings are in line with findings of Teo et al. showing that the effect of texture on food intake during lunch is not influenced by industrial processing level. Similarly to this study, an interaction effect was found. In the study by Teo et al., the least energy was consumed in the hard minimally processed meals and the most in the soft ultra-processed meals [30], which differed from this study’s findings as we observed lowest energy intake in the ultra-processed hard texture condition and highest in the ultra- processed soft texture condition. These differences can be explained by other factors that affect intake such as slightly higher palatability of some foods and the energy intake rate, which in the present study was highest for the ultra-processed soft condition. The effect on energy intake or energy intake rate strongly depends on the choice of food that was included in the design of the study.

The texture effect on food and energy intake is in line with previous studies that have shown that eating rate together with oro-sensory exposure duration, as two independent factors, drives the texture effect on food intake [22, 31]. In the present study, oro-sensory exposure duration was twice as long, and participants chewed twice as much on each bite of food, while the average bite size was smaller in the slow compared to the fast condition. The largest differences in eating behaviour were observed for the breakfast and dessert meals; this also translated in the largest difference in energy and food intake between the slow and fast conditions. Food intake per main meal, per study condition, cannot be compared separately as breakfast intake may influence intake at the subsequent meals. Additionally, meals may have differed in their satiety capacity due to differences in protein and fibre content, yet no difference in pre-meal appetite ratings were observed.

The hypothesized underlying mechanisms of texture affecting food intake are: (1) a direct effect, where oro-sensory exposure to food taste induces satiation through attenuated hedonic responses (sensory satiation), and (2) an indirect effect, where the slower eating rate increases the oro-sensory exposure duration of food. This then gives more time for satiation signals to be processed by the brain to induce feelings of satiation leading to meal cessation [19]. Moreover, chewing may be a (conscious and/or unconscious) ‘satiation cue’ independently of its effect on eating rate, as the dynamic feedback from oral and mechano-receptors in the oral cavity helps promote a faster onset of feelings of fullness [32]. The presented work did not investigate further downstream effects of eating rate on gastric and intestinal responses; little research has been done in this area and is an interesting opportunity for future studies.

In this study, we did not find a main effect of industrial processing level on energy intake that could not be explained by the eating behaviour characteristics. This is in contrast with findings of the previous study by Hall et al. that showed that people have higher energy intake when on a ultra-processed compared to unprocessed diet [8]. One possible explanation could be that the diets used in that study were equal in energy density, but not when excluding liquid calories from (soft)drinks [8]. Therefore, the ultra-processed diets contained more liquid calories compared to the unprocessed diets. As it is well known that liquid calories can be consumed fast without interoceptive cues on satiation, this could explain differences in energy intake between the two diets [19, 33]. Consequently, energy intake rate was also significantly greater during the ultra-processed meals (48 kcal/min) compared to the unprocessed meals (31 kcal/min) in the study of Hall [8]. In that study, the difference in daily energy intake between ultra- and unprocessed diets was 459 kcal. We found a difference in energy intake of 949 kcal between the hard,- and soft,- (ultra-) processed conditions but not between industrial processing levels. Therefore, the effects on energy intake in the study of Hall et al. are likely caused by the difference in energy intake rate rather than the difference in industrial processing level between the diets.

Strengths of the current study are the sustained effects of texture manipulations on daily energy intake and the detailed information about oral processing characteristics of the complete daily diets. Another strength of the study is the similarity in study meals per condition, in terms of palatability and energy density. Limitations of the present pilot trial are the small sample size leading to the underpower of the study to find significant difference of ≤ 10% between study conditions and the lack of variety within one study condition, i.e. multiple meals per study condition, and the controlled eating behaviour research unit environment, which may limit the ecological validity of the study. Additionally, two of the participants made at least some correct assumptions about the goal of the study. Given the visceral nature of the manipulation, this is difficult to prevent, whether or not being aware of the UPF level or texture properties of the food influences food or kcal intake should be considered in future studies investigating food intake of UPF foods differing in texture or eating rate. Based on this pilot trial, we can argue that there is large variability in food properties from the unprocessed to ultra-processed food categories that may drive or moderate energy and food intake. Therefore, excluding (ultra-) processed foods completely from the diet or food supply is not advisable as this may lead to a decrease in food security [9]. Especially, ultra-processed foods are commonly consumed in countries worldwide, ranging from 60% of the calories derived from ultra-processed foods in the average US diet, to 50–60% in Germany and the Netherlands, to ~ 40% in the average Australian diet and to 20–30% in France, Brazil and Spain [34–39]. Instead, there should be more emphasis on the energy intake rates of new food designs to prevent overconsumption. High energy dense foods should have textures that requires a longer oral processing and should not be consumed too quickly.

Although energy intake rate increases with the degree of processing of foods, within each NOVA classification there are food items with a broad range of eating rates [15]. Results of observational studies using the NOVA classification may be confounded when not differentiating between high and low energy intake rate foods. It is at least likely that part of the found associations between (ultra-)processed foods and health outcomes can be ascribed to food texture and energy density. Therefore, future observational studies should include energy density and energy intake rates as confounders in models predicting associations between NOVA groups and health outcomes. Additionally, future experimental studies may investigate how textural manipulations slow down energy intake rates and lead to sustained moderation in energy intake.

## Conclusion

This pilot trial shows that harder food textures reduce daily food and energy intake of (ultra-) processed foods in healthy weight people, when meals are matched for energy density and palatability. This shows that within a NOVA class there is variability in food properties that affect food and energy intake beyond industrial food processing. However, findings should be interpreted with precaution considering the limited sample size. Classifying foods based on energy intake rate would be an effective strategy to prevent food overconsumption. Energy intake rate could be used both for public health messages and to reformulate foods through innovate food processing designs to prevent overconsumption of calories. By doing so, public health programmes and food industry could help make progress towards better prevention of passive food overconsumption, preventing overweight and obesity.

## Supplementary Information

Below is the link to the electronic supplementary material.

Supplementary file1 (DOCX 22365 KB)

## Data Availability

Data is made available upon request.

## Abbreviations

OSE: Oro-sensory exposure
BMI: Body Mass Index
VAS: Visual analogue scale
DEBQ: Dutch Eating Behaviour Questionnaire

## References

[1] World Health Organization TUN. Obesity and overweight. https://www.who.int/news-room/fact-sheets/detail/obesity-and-overweight. Accessed in Jan 2021

[2] Monteiro CA, Moubarac JC, Cannon G, Ng SW, Popkin B (2013). Ultra-processed products are becoming dominant in the global food system. Obes Rev.

[3] Juul F, Hemmingsson E (2015). Trends in consumption of ultra-processed foods and obesity in Sweden between 1960 and 2010. Public Health Nutr.

[4] Capozzi F, Magkos F, Fava F, Milani GP, Agostoni C, Astrup A, Saguy IS (2021). A multidisciplinary perspective of ultra-processed foods and associated food processing technologies: a view of the sustainable road ahead. Nutrients.

[5] Karmas E, Harris RS (2012). Nutritional evaluation of food processing.

[6] Monteiro CA, Cannon G, Lawrence M, Costa Louzada Md, Pereira Machado P (2019). Ultra-processed foods, diet quality, and health using the NOVA classification system.

[7] Askari M, Heshmati J, Shahinfar H, Tripathi N, Daneshzad E (2020). Ultra-processed food and the risk of overweight and obesity: a systematic review and meta-analysis of observational studies. Int J Obes (Lond).

[8] Hall KD, Ayuketah A, Brychta R, Cai HY, Cassimatis T, Chen KY, Chung ST, Costa E, Courville A, Darcey V, Fletcher LA, Forde CG, Gharib AM, Guo J, Howard R, Joseph PV, McGehee S, Ouwerkerk R, Raisinger K, Rozga I, Stagliano M, Walter M, Walter PJ, Yang S, Zhou MG (2019). Ultra-processed diets cause excess calorie intake and weight gain: an inpatient randomized controlled trial of Ad libitum food intake (vol 30, pg 67, 2019). Cell Metab.

[9] Tobias DK, Hall KD (2021). Eliminate or reformulate ultra-processed foods? Biological mechanisms matter. Cell Metab.

[10] Poti JM, Braga B, Qin B (2017). Ultra-processed food intake and obesity: what really matters for health-processing or nutrient content?. Curr Obes Rep.

[11] Fardet A (2016). Minimally processed foods are more satiating and less hyperglycemic than ultra-processed foods: a preliminary study with 98 ready-to-eat foods. Food Funct.

[12] Rolls BJ, Cunningham PM, Diktas HE (2020). Properties of ultraprocessed foods that can drive excess intake. Nutr Today.

[13] Krop EM, Hetherington MM, Nekitsing C, Miquel S, Postelnicu L, Sarkar A (2018). Influence of oral processing on appetite and food intake—a systematic review and meta-analysis. Appetite.

[14] Robinson E, Almiron-Roig E, Rutters F, de Graaf C, Forde CG, Tudur Smith C, Nolan SJ, Jebb SA (2014). A systematic review and meta-analysis examining the effect of eating rate on energy intake and hunger. Am J Clin Nutr.

[15] Forde CG, Mars M, de Graaf K (2020). Ultra-processing or oral processing? A role for energy density and eating rate in moderating energy intake from processed foods. Curr Dev Nutr.

[16] Appleton KM, Newbury A, Almiron-Roig E, Yeomans MR, Brunstrom JM, de Graaf K, Geurts L, Kildegaard H, Vinoy S (2021). Sensory and physical characteristics of foods that impact food intake without affecting acceptability: systematic review and meta-analyses. Obes Rev.

[17] Rolls BJ (2009). The relationship between dietary energy density and energy intake. Physiol Behav.

[18] Bolhuis DP, Forde CG, Cheng Y, Xu H, Martin N, de Graaf C (2014). Slow food: sustained impact of harder foods on the reduction in energy intake over the course of the day. PLoS ONE.

[19] Lasschuijt MP, de Graaf K, Mars M (2021). Effects of oro-sensory exposure on satiation and underlying neurophysiological mechanisms-what do we know so far?. Nutrients.

[20] Bolhuis DP, Forde CG (2020). Application of food texture to moderate oral processing behaviors and energy intake. Trends Food Sci Technol.

[21] Van Strien T (2005). Nederlandse vragenlijst voor eetgedrag (NVE). Handeleiding. (Dutch eating behavior questionnaire Manual).

[22] Bolhuis DP, Lakemond CM, de Wijk RA, Luning PA, Graaf C (2011). Both longer oral sensory exposure to and higher intensity of saltiness decrease ad libitum food intake in healthy normal-weight men. J Nutr.

[23] Lasschuijt MP, Mars M, Stieger M, Miquel-Kergoat S, de Graaf C, Smeets P (2017). Comparison of oro-sensory exposure duration and intensity manipulations on satiation. Physiol Behav.

[24] Laursen RP, Dalskov SM, Damsgaard CT, Ritz C (2014). Back-transformation of treatment differences-an approximate method. Eur J Clin Nutr.

[25] Forde CG, van Kuijk N, Thaler T, de Graaf C, Martin N (2013). Texture and savoury taste influences on food intake in a realistic hot lunch time meal. Appetite.

[26] Hogenkamp PS, Schioth HB (2013). Effect of oral processing behaviour on food intake and satiety. Trends Food Sci Technol.

[27] Zijlstra N, Mars M, de Wijk RA, Westerterp-Plantenga MS, de Graaf C (2008). The effect of viscosity on ad libitum food intake. Int J Obes.

[28] Zijlstra N, Mars M, Stafleu A, de Graaf C (2010). The effect of texture differences on satiation in 3 pairs of solid foods. Appetite.

[29] Pritchard SJ, Davidson I, Jones J, Bannerman E (2014). A randomised trial of the impact of energy density and texture of a meal on food and energy intake, satiation, satiety, appetite and palatability responses in healthy adults. Clin Nutr.

[30] Teo PS, Lim AJ, Goh AT, Choy JYM, McCrickerd K, Forde CG (2022). Texture-based differences in eating rate influence energy intake for minimally processed and ultra-processed meals. Am J Clin Nutr.

[31] Weijzen PL, Smeets PA, de Graaf C (2009). Sip size of orangeade: effects on intake and sensory-specific satiation. Br J Nutr.

[32] Hollis JH (2018). The effect of mastication on food intake, satiety and body weight. Physiol Behav.

[33] DiMeglio D, Mattes R (1999). Liquid versus solid carbohydrate (CHO): effects on food intake and body weight. Faseb J.

[34] Slimani N, Deharveng G, Southgate DA, Biessy C, Chajes V, van Bakel MM, Boutron-Ruault MC, McTaggart A, Grioni S, Verkaik-Kloosterman J, Huybrechts I, Amiano P, Jenab M, Vignat J, Bouckaert K, Casagrande C, Ferrari P, Zourna P, Trichopoulou A, Wirfalt E, Johansson G, Rohrmann S, Illner AK, Barricarte A, Rodriguez L, Touvier M, Niravong M, Mulligan A, Crowe F, Ocke MC, van der Schouw YT, Bendinelli B, Lauria C, Brustad M, Hjartaker A, Tjonneland A, Jensen AM, Riboli E, Bingham S (2009). Contribution of highly industrially processed foods to the nutrient intakes and patterns of middle-aged populations in the European Prospective Investigation into cancer and nutrition study. Eur J Clin Nutr.

[35] Wang L, Martínez Steele E, Du M, Pomeranz JL, O’Connor LE, Herrick KA, Luo H, Zhang X, Mozaffarian D, Zhang FF (2021). Trends in consumption of ultraprocessed foods among US youths aged 2–19 years, 1999–2018. JAMA.

[36] Machado PP, Steele EM, Levy RB, da Costa Louzada ML, Rangan A, Woods J, Gill T, Scrinis G, Monteiro CA (2020). Ultra-processed food consumption and obesity in the Australian adult population. Nutr Diabetes.

[37] Llavero-Valero M, Escalada-San Martin J, Martinez-Gonzalez MA, Basterra-Gortari FJ, de la Fuente-Arrillaga C, Bes-Rastrollo M (2021). Ultra-processed foods and type-2 diabetes risk in the SUN project: a prospective cohort study. Clin Nutr.

[38] Calixto Andrade G, Julia C, Deschamps V, Srour B, Hercberg S, Kesse-Guyot E, Allès B, Chazelas E, Deschasaux M, Touvier M, Augusto Monteiro C, Bertazzi Levy R (2021). Consumption of ultra-processed food and its association with sociodemographic characteristics and diet quality in a representative sample of French adults. Nutrients.

[39] Vellinga RE, van Bakel M, Biesbroek S, Toxopeus IB, de Valk E, Hollander A, van’t Veer P, Temme EHM (2022). Evaluation of foods, drinks and diets in the Netherlands according to the degree of processing for nutritional quality, environmental impact and food costs. BMC Public Health.

