# Supplementary material for: Speed limits: the effects of industrial food processing and food texture on daily energy intake and eating behaviour in healthy adults
Source: Eur J Nutr. 2023 Jul 14;62(7):2949–62. doi: 10.1007/s00394-023-03202-z (PMC10469122; doi:10.1007/s00394-023-03202-z)
Supplement: Supplementary file 1 — Supplementary file1 (DOCX 22365 KB) [file 394_2023_3202_MOESM1_ESM.docx]

# Speed limits: The effects of industrial food processing and food texture on daily energy intake and eating behaviour in healthy adults

*Running title: Food texture, level of food processing and energy intake*

**Marlou Lasschuijt^1^, Guido Camps^1^, Monica Mars^1^, Els Siebelink^1^, Kees de Graaf^1^, Dieuwerke Bolhuis^2^**

^1^Division of Human Nutrition and Health, Wageningen University & Research, The Netherlands

^2^ Food Quality and Design Group, Wageningen University & Research, The Netherlands

# Supplement 1- Study meals per condition including NOVA classification

The menu’s shown and described below depict the meals as given to the participants. The macronutrient and energy content of the meals can be found in Supplement 2.

**Unprocessed, Hard texture- Menu**

**Breakfast**

| Ingredients | Weight% of total weight (1888 gram) | Kcal%  of total (882 kcal) | NOVA class | Number of ingredients incl. additives | Product type or brand |
| --- | --- | --- | --- | --- | --- |
| water | 33.4 | 0.0 | 1 | - | Tap water |
| apple with peel | 28.9 | 34.1 | 1 | - | Pink lady |
| banana | 17.6 | 34.8 | 1 | - | - |
| strawberry | 3.2 | 1.9 | 1 | - | - |
| cherry | 2.2 | 2.6 | 1 | - | - |
| blueberry | 1.7 | 1.9 | 1 | - | - |
| blue grapes | 6.3 | 10.1 | 1 | - | - |
| white grapes | 6.3 | 10.2 | 1 | - | - |
| linseed | 0.4 | 4.3 | 1 | - | Broken seeds |


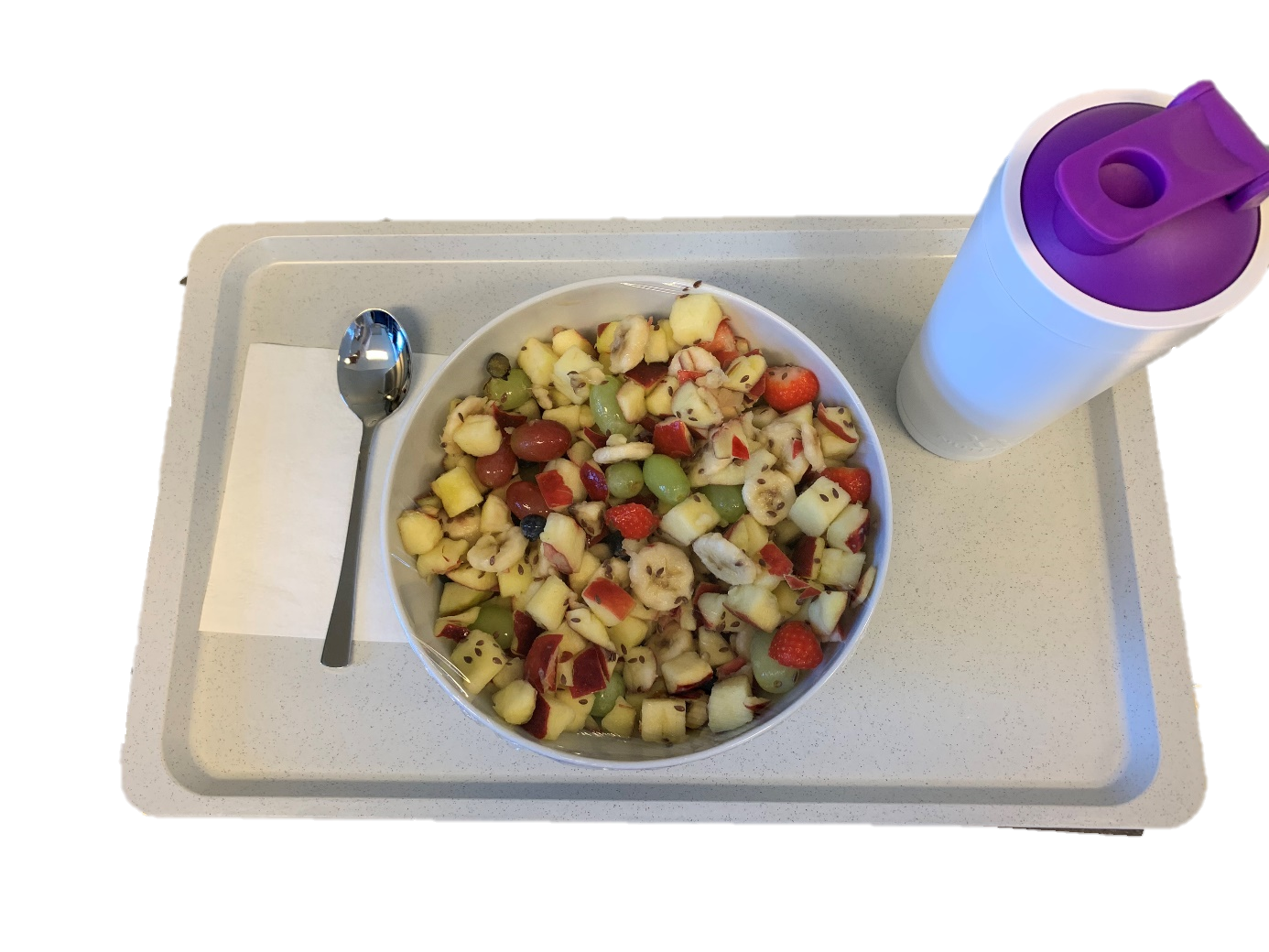


**Morning Snack**

| Ingredients | Weight% of total weight (90 gram) | Kcal%  of total (50 kcal) | NOVA class | Number of ingredients incl. additives | Product type or brand |
| --- | --- | --- | --- | --- | --- |
| Apple with peel with fresh lemon juice | 100 | 100 | 1 | 2 | Elstar |

**Lunch**

| Ingredients | Weight% of total weight (1078 gram) | Kcal%  of total (1608 kcal) | NOVA class | Number of ingredients incl. additives | Product type or brand |
| --- | --- | --- | --- | --- | --- |
| chicken filet | 22.3 | 23.6 | 1 | - | - |
| carrot steamed | 18.6 | 4.0 | 1 | - | - |
| tomato fresh, steamed | 14.8 | 3.1 | 1 | - | - |
| tagliatelle fresh, boiled | 10.4 | 20.0 | 1 | 2 | Albert Heijn fresh tagliatelle all'uovo |
| zucchini steamed | 9.3 | 1.2 | 1 | - | - |
| water | 8.2 | 0.0 | 1 | - | - |
| sunflower oil | 5.6 | 33.5 | 2 | 1 | Albert Heijn |
| onion steamed | 3.7 | 0.9 | 1 | - | - |
| Flower honey | 3.7 | 8.1 | 2 | - | Albert Heijn, Breitsamer |
| creme fraiche | 2.8 | 5.5 | 1 | 1 | Albert Heijn, Fresh creme fraiche |
| basil fresh | 0.5 | 0.1 | 1 | - | - |
| garlic raw | 0.2 | 0.2 | 1 | - | - |


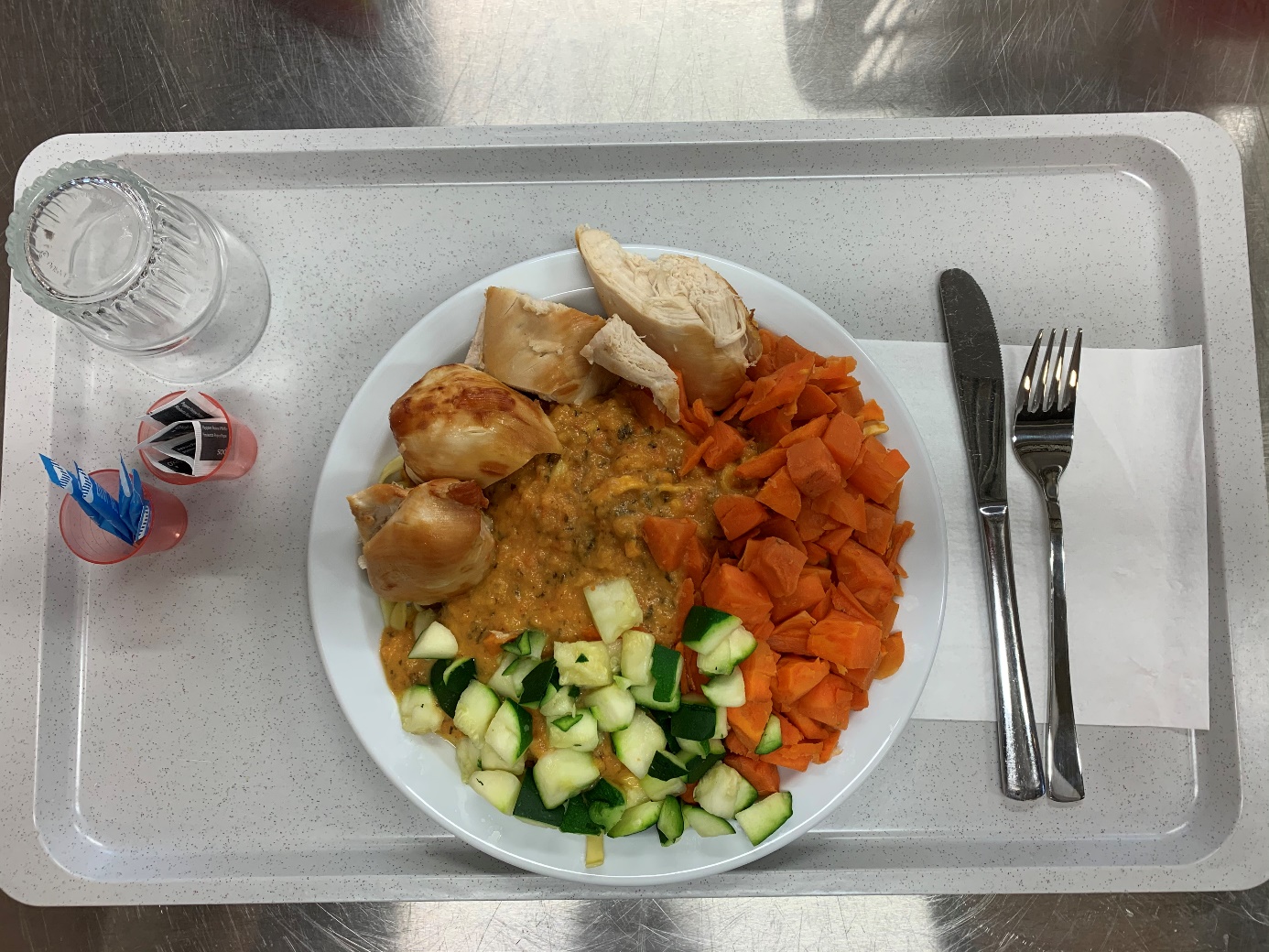


**Afternoon Snack**

| Ingredients | Weight% of total weight (50 gram) | Kcal%  of total (170 kcal) | NOVA class | Number of ingredients incl. additives | Product type or brand |
| --- | --- | --- | --- | --- | --- |
| Dried mango pieces | 100 | 100 | 1 | 1 | Albert Heijn, organic |

**Dinner**

| Ingredients | Weight% of total weight (831 gram) | Kcal%  of total (685 kcal) | NOVA class | Number of ingredients incl. additives | Product type or brand |
| --- | --- | --- | --- | --- | --- |
| potato with skin boiled | 36.1 | 32.4 | 1 | - | - |
| green beans fresh boiled | 36.1 | 10.9 | 1 | - | - |
| Pork filet | 24.1 | 45.8 | 1 | - | - |
|  | *Sauce:* |  |  |  |  |
| Flower honey | 2.4 | 9.5 | 2 |  | Albert Heijn, Breitsamer |
| Onion baked | 0.4 | 0.3 | 1 | - | - |
| chili pepper raw | 0.3 | 0.1 | 1 | - | - |
| Dutch shrimps cooked | 0.2 | 0.3 | 2 | - | Albert Heijn |
| Lemon juice fresh | 0.2 | 0.1 | 1 | - | - |
| Coconut meat | 0.1 | 0.4 | 1 | 1 | Albert Heijn sun dried coconut pieces |
| garlic raw | 0.0 | 0.0 | 1 | - | - |


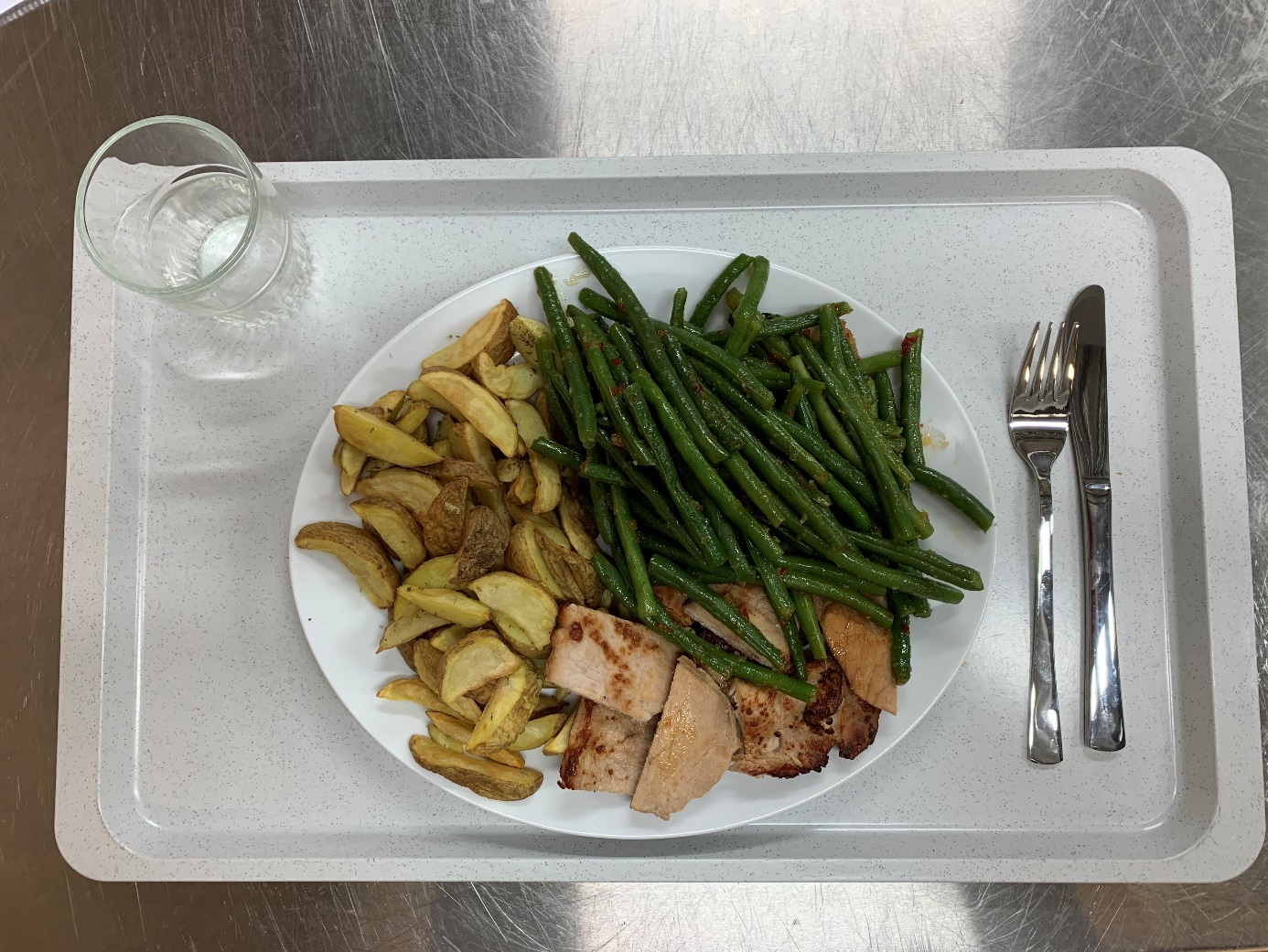


**Dessert**

| Ingredients | Weight% of total weight (340 gram) | Kcal%  of total  (1042 kcal) | NOVA class | Number of ingredients incl. additives | Product type or brand |
| --- | --- | --- | --- | --- | --- |
| figs dried | 95 | 89.8 | 1 | 1 | Albert Heijn |
| Almonds unsalted | 5 | 10.2 | 1 | - | - |


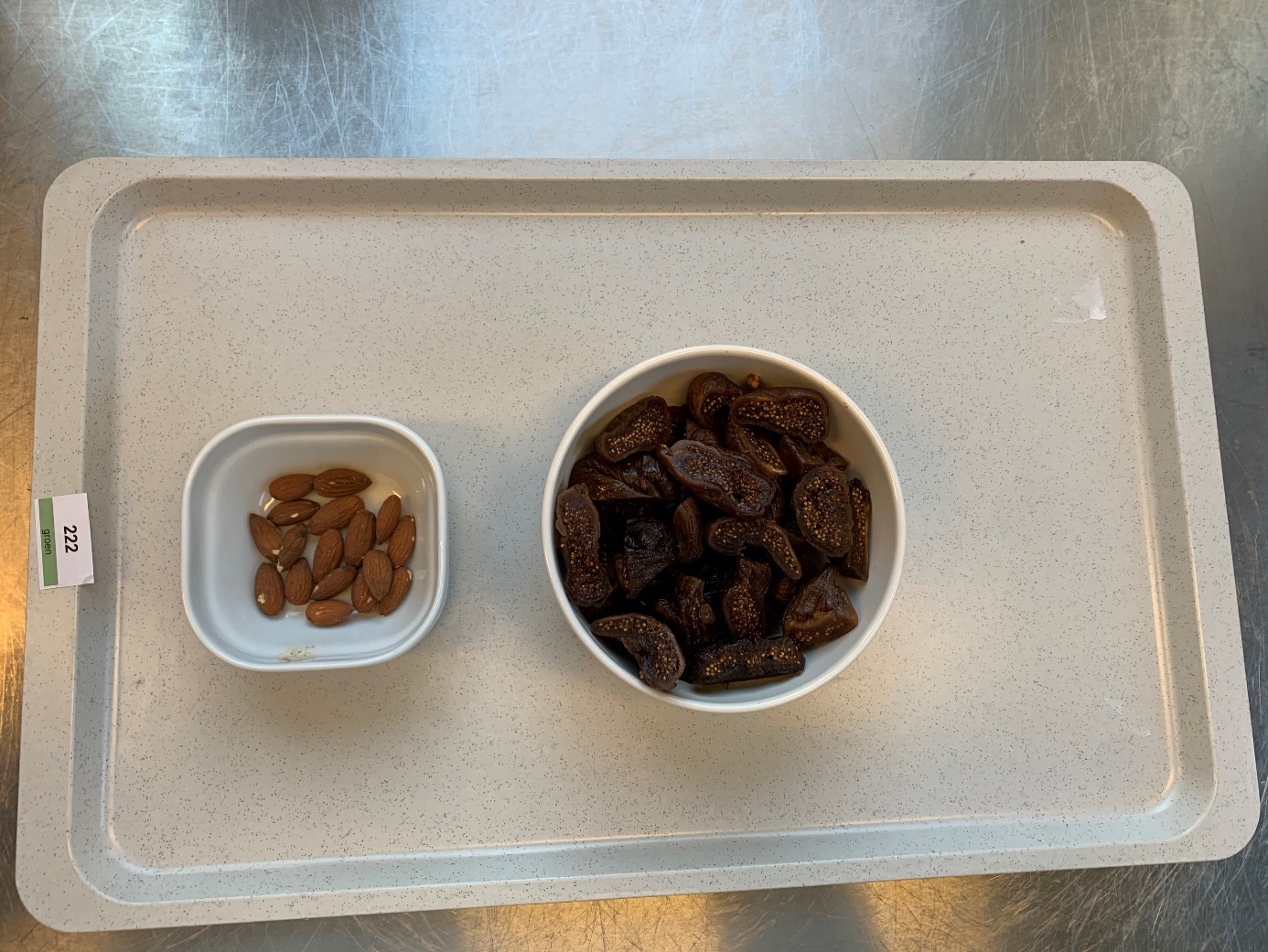
**Unprocessed, Soft Texture- Menu**

**Breakfast**

| Ingredients | Weight% of total weight (1888 gram) | Kcal%  of total (882 kcal) | NOVA class | Number of ingredients incl. additives | Product type or brand |
| --- | --- | --- | --- | --- | --- |
| water | 33.4 | 0.0 | 1 | - | Tap water |
| apple with peel | 28.9 | 34.1 | 1 | - | Pink lady |
| banana | 17.6 | 34.8 | 1 | - | - |
| blue grape | 6.3 | 10.1 | 1 | - | - |
| white grape | 6.3 | 10.2 | 1 | - | - |
| strawberry | 3.2 | 1.9 | 1 | - | - |
| cherry | 2.2 | 2.6 | 1 | - | - |
| blueberry | 1.7 | 1.9 | 1 | - | - |
| linseed | 0.4 | 4.3 | 1 | - | Broken seeds |


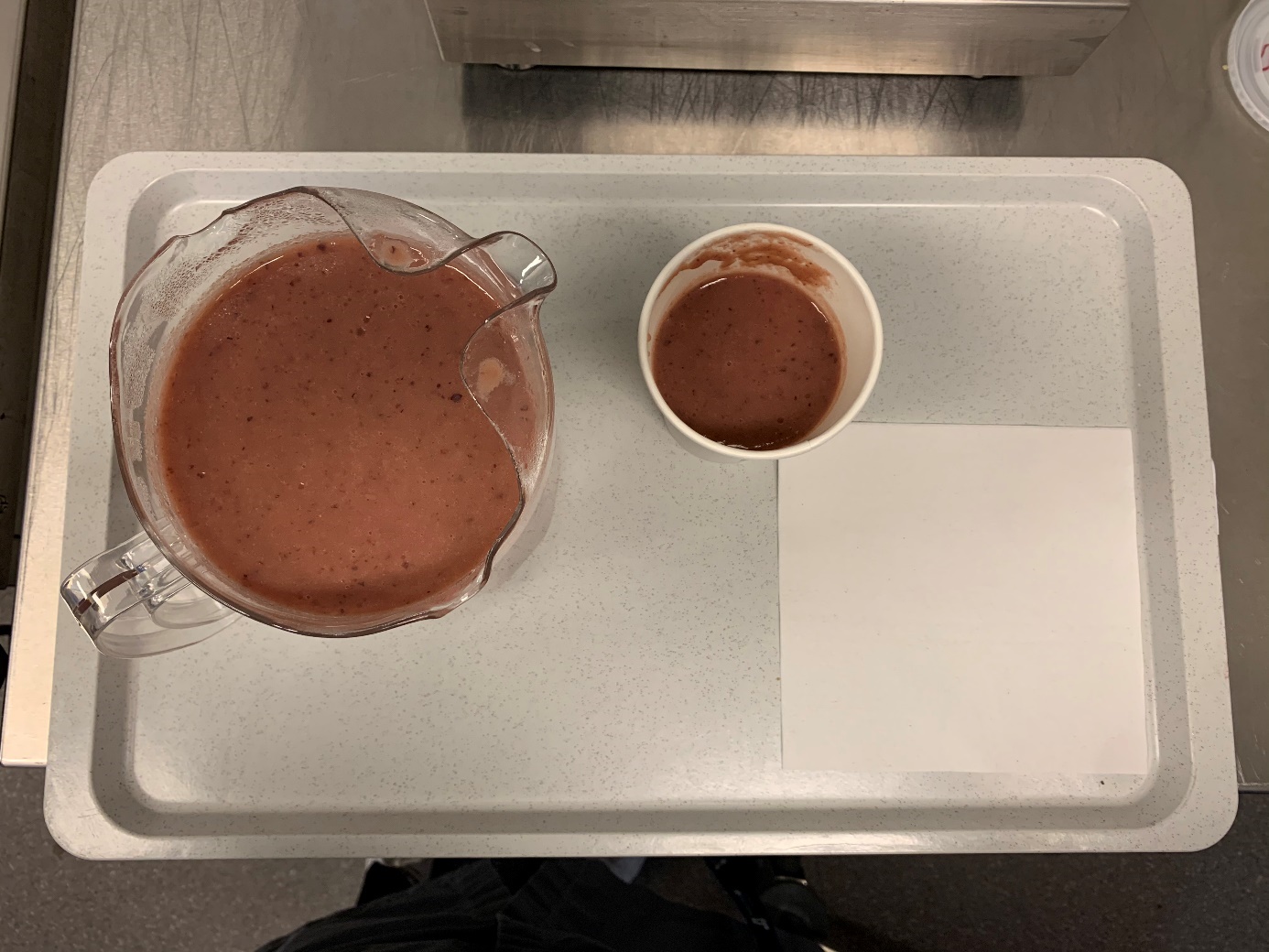


**Morning Snack**

| Ingredients | Weight% of total weight (90 gram) | Kcal%  of total (43 kcal) | NOVA class | Number of ingredients incl. additives | Product type or brand |
| --- | --- | --- | --- | --- | --- |
| apple sauce no added sugar | 100 | 100 | 1 | 1 | Albert Heijn  organic 0% sugar |

**Lunch**

| Ingredients | Weight% of total weight (1078 gram) | Kcal%  of total  (1608 kcal) | NOVA class | Number of ingredients incl. additives | Product type or brand |
| --- | --- | --- | --- | --- | --- |
| chicken filet prepared | 22.3 | 23.6 | 1 | - | - |
| Carrot, steamed | 18.6 | 4.0 | 1 | - | - |
| tomato fresh, steamed | 14.8 | 3.1 | 1 | - | - |
| tagliatelle fresh, boiled | 10.4 | 20.0 | 1 | 2 | Albert Heijn fresh tagliatelle all'uovo |
| zucchini steamed | 9.3 | 1.2 | 1 | - | - |
| water | 8.2 | 0.0 | 1 | - | - |
| sunflower oil | 5.6 | 33.5 | 2 | 1 | Albert Heijn |
| onion steamed | 3.7 | 0.9 | 1 | - | - |
| Flower honey | 3.7 | 8.1 | 2 | - | Albert Heijn, Breitsamer |
| creme fraiche | 2.8 | 5.5 | 1 | 1 | Albert Heijn, Fresh creme fraiche |
| garlic raw | 0.2 | 0.2 | 1 | - |  |
| basil fresh | 0.5 | 0.1 | 1 | - | - |


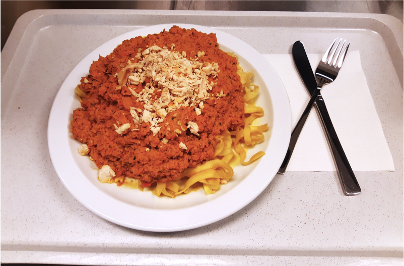
**Afternoon Snack**

| Ingredients | Weight% of total weight (50 gram) | Kcal%  of total (163 kcal) | NOVA class | Number of ingredients incl. additives | Product type or brand |
| --- | --- | --- | --- | --- | --- |
| Dried raisins | 100 | 100 | 2 | 2 | Albert Heijn  basic |

**Dinner**

| Ingredients | Weight% of total weight (831 gram) | Kcal%  of total  (731 kcal) | NOVA class | Number of ingredients incl. additives | Product type or brand |
| --- | --- | --- | --- | --- | --- |
| green beans steamed | 36.1 | 10.3 | 1 | - | - |
| potato without skin boiled | 36.1 | 33.2 | 1 | - | - |
| egg boiled | 18.1 | 26.3 | 1 | - | - |
| creme fraiche | 6.0 | 20.1 | 1 | 1 | Albert Heijn |
|  | *Sauce:* |  |  |  |  |
| Flower honey | 2.4 | 8.9 | 2 | 2 | Albert Heijn, Breitsamer |
| Coconut meat | 0.1 | 0.4 | 1 | - | - |
| Lemon juice fresh | 0.2 | 0.1 | 1 | - | - |
| chili pepper raw | 0.3 | 0.1 | 1 |  |  |
| Dutch shrimps cooked | 0.2 | 0.3 | 2 | 5 | - |
| onion baked | 0.4 | 0.3 | 1 | - | - |
| garlic raw | 0.0 | 0.0 | 1 | - | - |


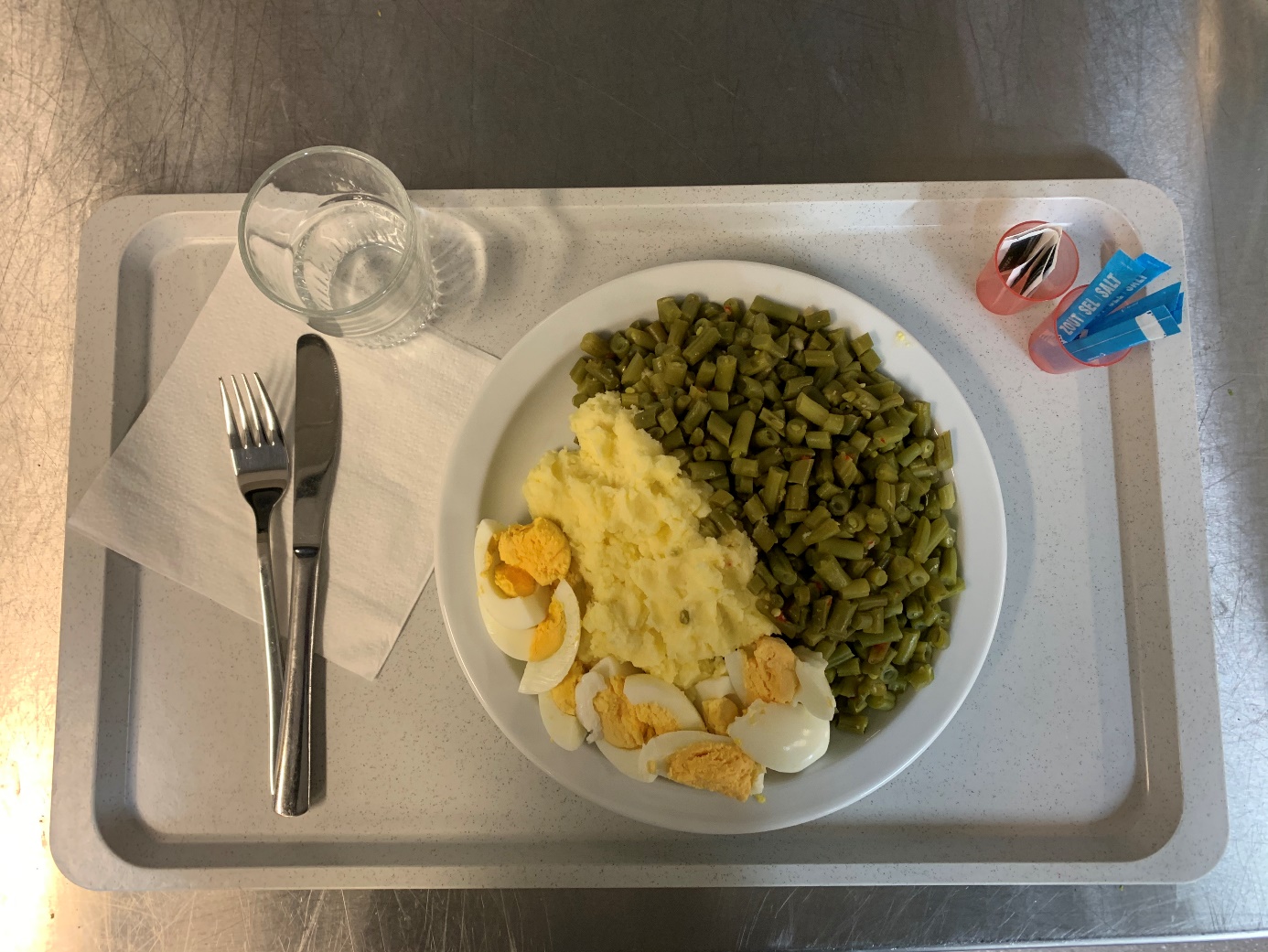


**Dessert**

| Ingredients | Weight% of total weight (350 gram) | Kcal%  of total (1040 kcal) | NOVA class | Number of ingredients incl. additives | Product type or brand |
| --- | --- | --- | --- | --- | --- |
| curd | 60.0 | 18.2 | 1 | 1 | Albert Heijn, mild dutch ‘ hangop’ |
| pecan nuts unsalted | 28.6 | 69.3 | 1 | - | - |
| flower  honey | 11.4 | 12.5 | 2 | 2 | Albert Heijn, Breitsamer |


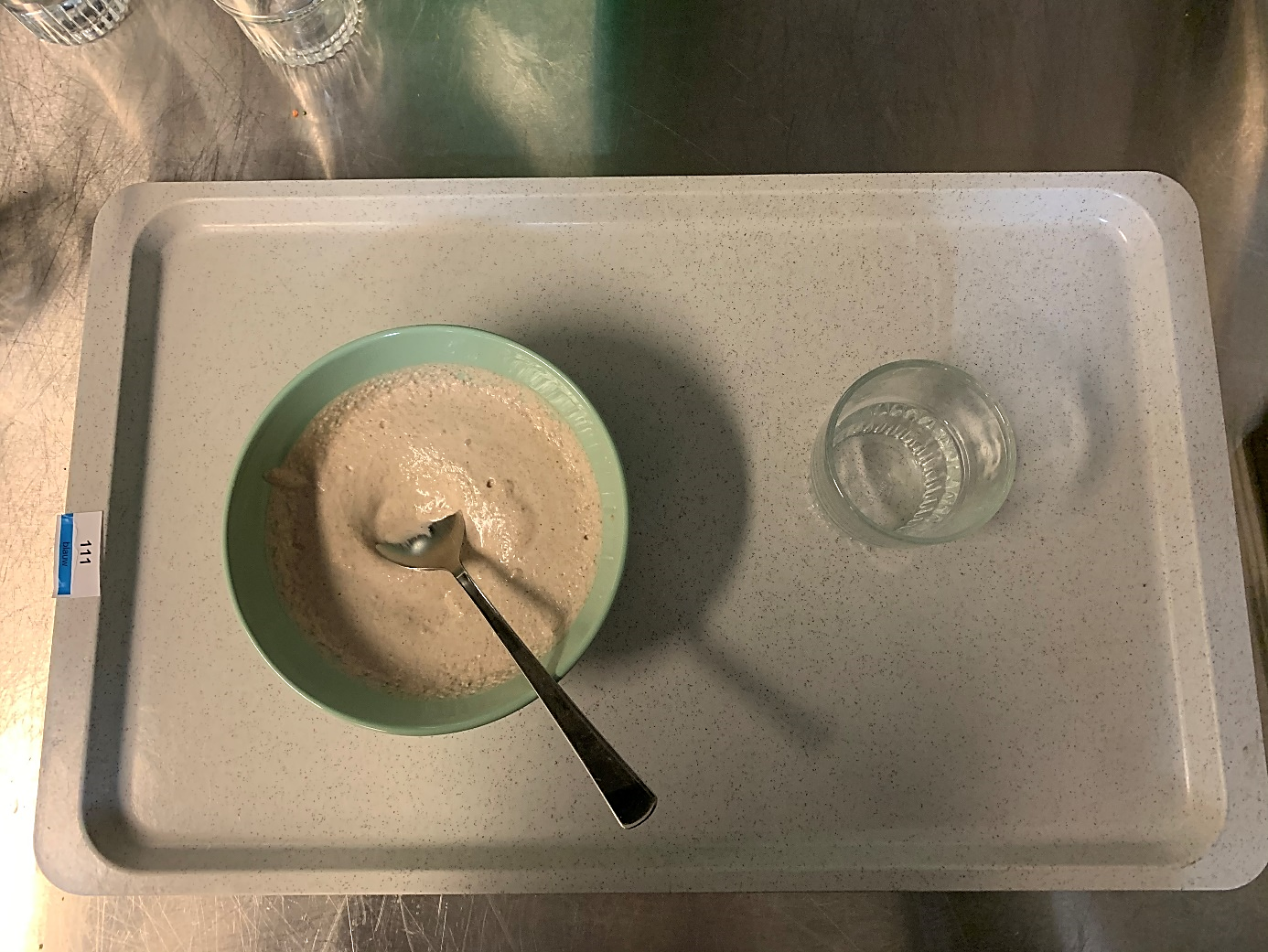
**(ultra-)processed, Hard texture- Menu**

**Breakfast**

| Ingredients | Weight% of total weight (1880 gram) | Kcal%  of total (820 kcal) | NOVA class | Number of ingredients incl. additives | Product type or brand |
| --- | --- | --- | --- | --- | --- |
| fruit salad on syrup canned | 53.2 | 74.4 | 3 | 9 | Albert Heijn basic |
| water | 28.2 | 0.0 | 1 | 1 | Tap water |
| half peaches on syrup canned | 18.6 | 25.6 | 3 | 5 | Albert Heijn basic, El Greco |


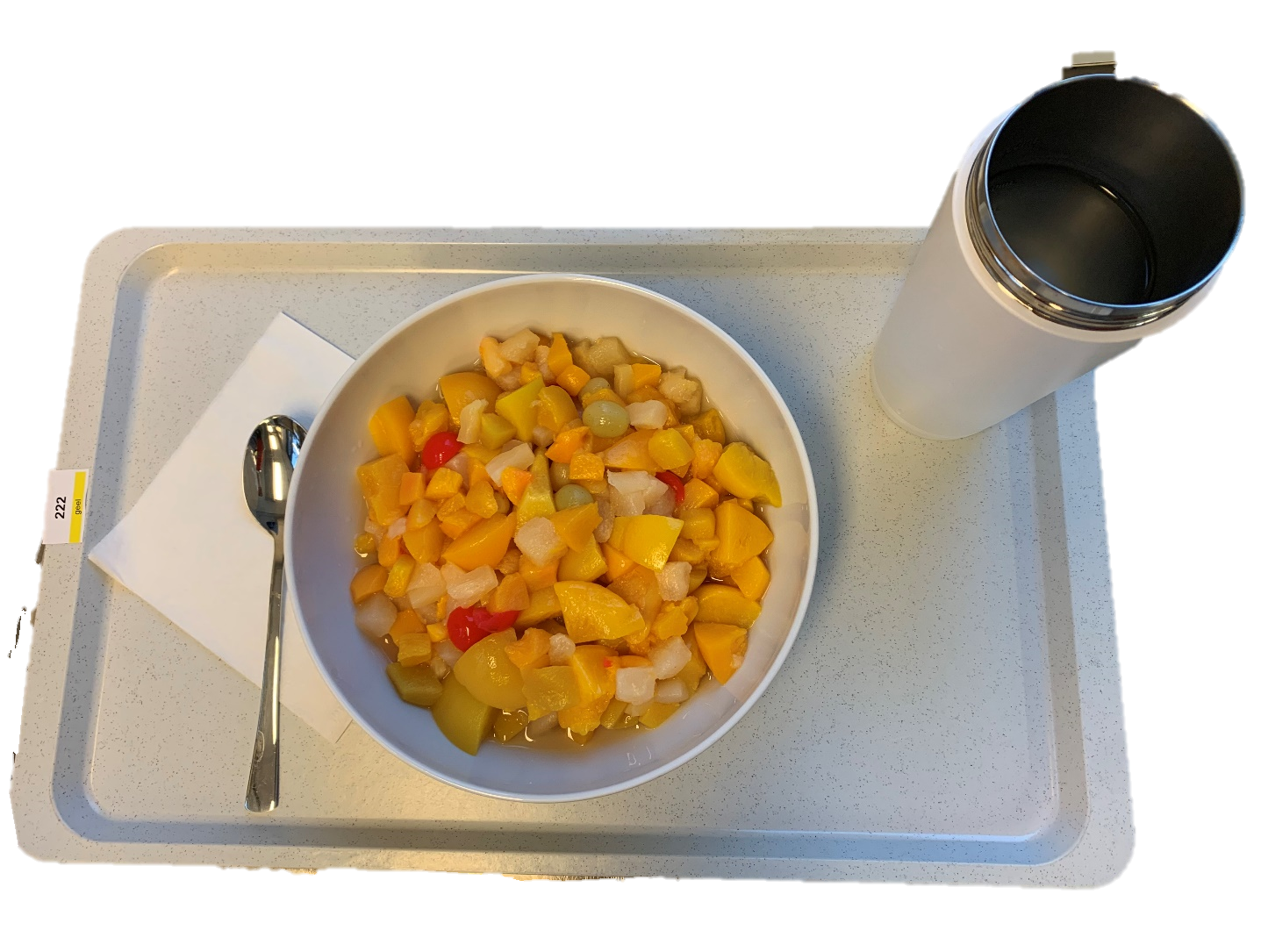


**Snack**

| Ingredients | Weight% of total weight (90 gram) | Kcal%  of total (50 kcal) | NOVA class | Number of ingredients incl. additives | Product type or brand |
| --- | --- | --- | --- | --- | --- |
| Apple with peel pre-cut and packed | 100 | 100 | 2 | 2 | Elstar |

**Lunch**

| Ingredients | Weight% of total weight (1260 gram) | Kcal%  of total (1597 kcal) | NOVA class | Number of ingredients incl. additives | Product type or brand |
| --- | --- | --- | --- | --- | --- |
| tortellini boiled | 34.9 | 42.7 | 4 | 20 | Tortellini al gusto carne, grand’ Italia |
| pasta sauce canned | 27.4 | 10.8 | 3 | 6 | Bertolli pasta sauce basilico |
| carrot steamed | 15.9 | 4.0 | 1 | 1 | - |
| Full fat cheese | 13.9 | 41.3 | 3 | 5 | Goudse mature cheese 48+ |
| zucchini steamed | 7.9 | 1.2 | 1 | 1 | - |


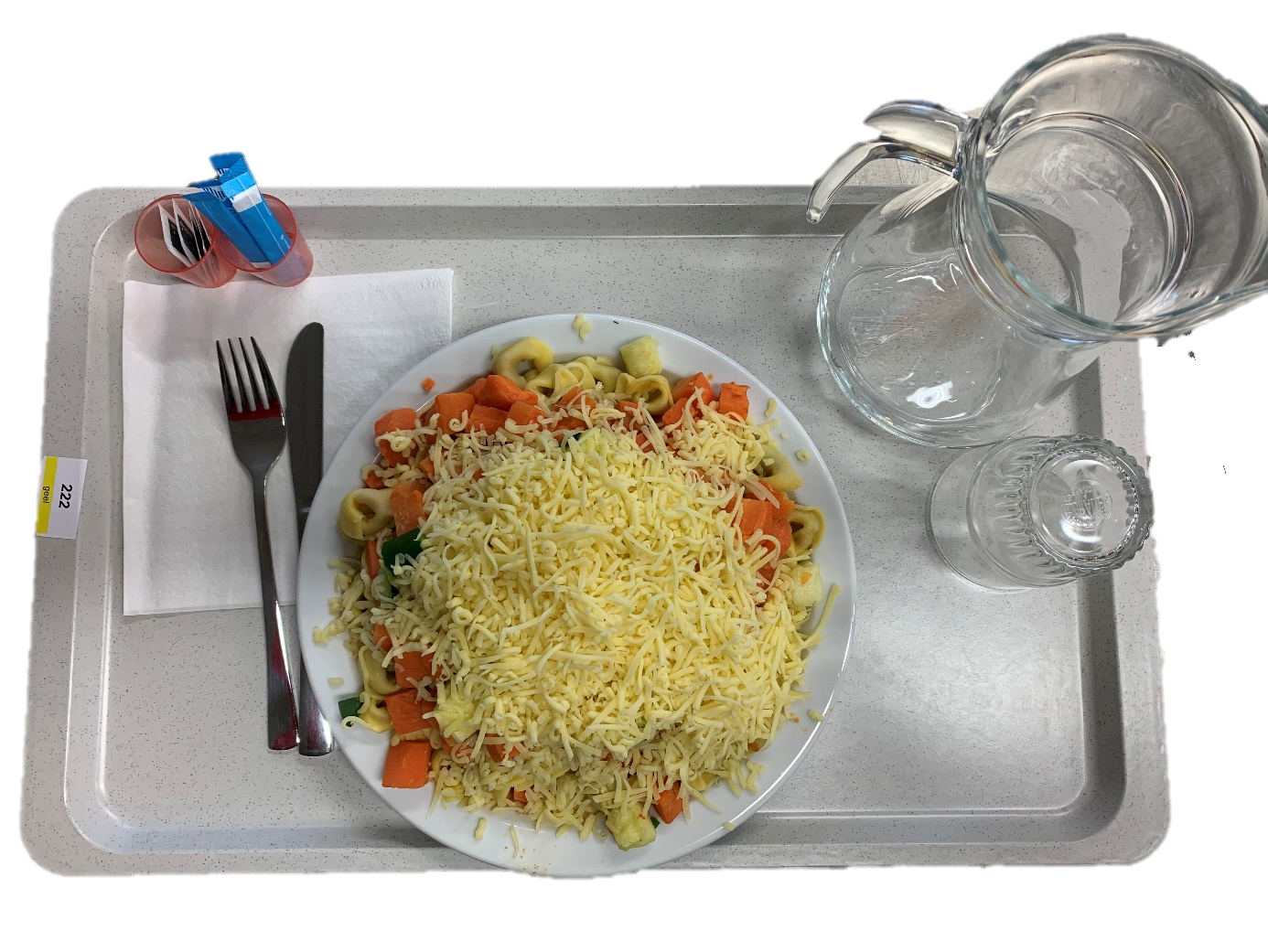


**Afternoon Snack**

| Ingredients | Weight% of total weight (50 gram) | Kcal%  of total (195 kcal) | NOVA class | Number of ingredients incl. additives | Product type or brand |
| --- | --- | --- | --- | --- | --- |
| Muesli bar hazelnut | 100 | 100 | 4 | 16 | Albert Heijn  Muesli hazelnut bar |

**Dinner**

| Ingredients | Weight%  of total weight (820 gram) | Kcal%  of total (769 kcal) | NOVA class | Number of ingredients incl. additives | Product type or brand |
| --- | --- | --- | --- | --- | --- |
| green beans steamed | 36.6 | 9.8 | 1 | 0 | - |
| potato pieces baked | 36.6 | 35.1 | 3 | 6 | Albert Heijn Potato parts with herbs of the province |
| chicken schnitzel crispy prepared | 20.7 | 52.0 | 4 | 15 | Albert Heijn crispy chickenfiletschnitzel |
| AH sajoer | 6.1 | 3.1 | 4 | 27 | Albert Heijn Boemboe sajoer boontjes |


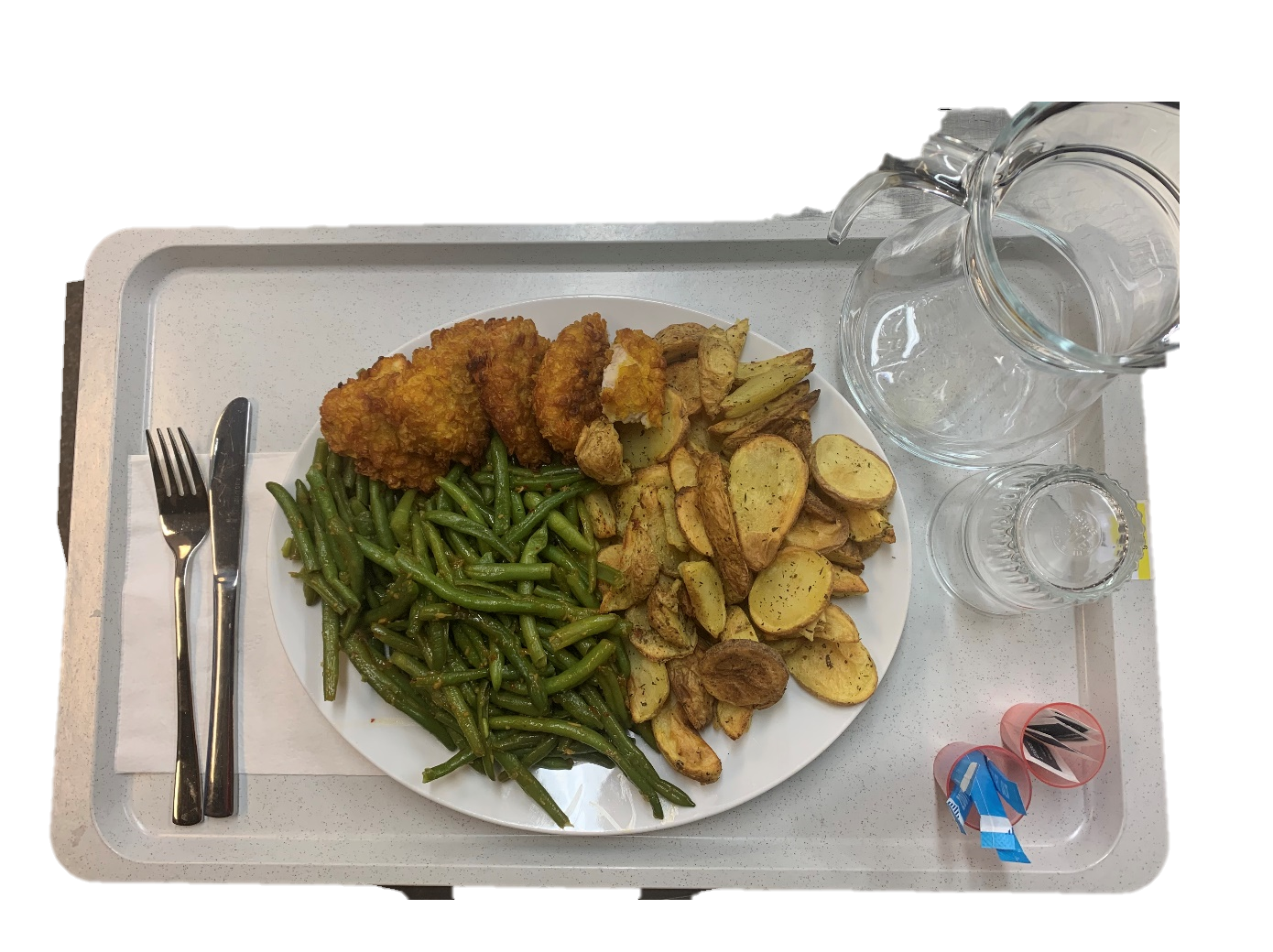


**Dessert**

| Ingredients | Weight% of total weight (340 gram) | Kcal%  of total (1074 kcal) | NOVA class | Number of ingredients incl. additives | Product type or brand |
| --- | --- | --- | --- | --- | --- |
| Fig bread with almonds | 100 | 100 | 1 | 2 | Ana Luz figbread with almonds |


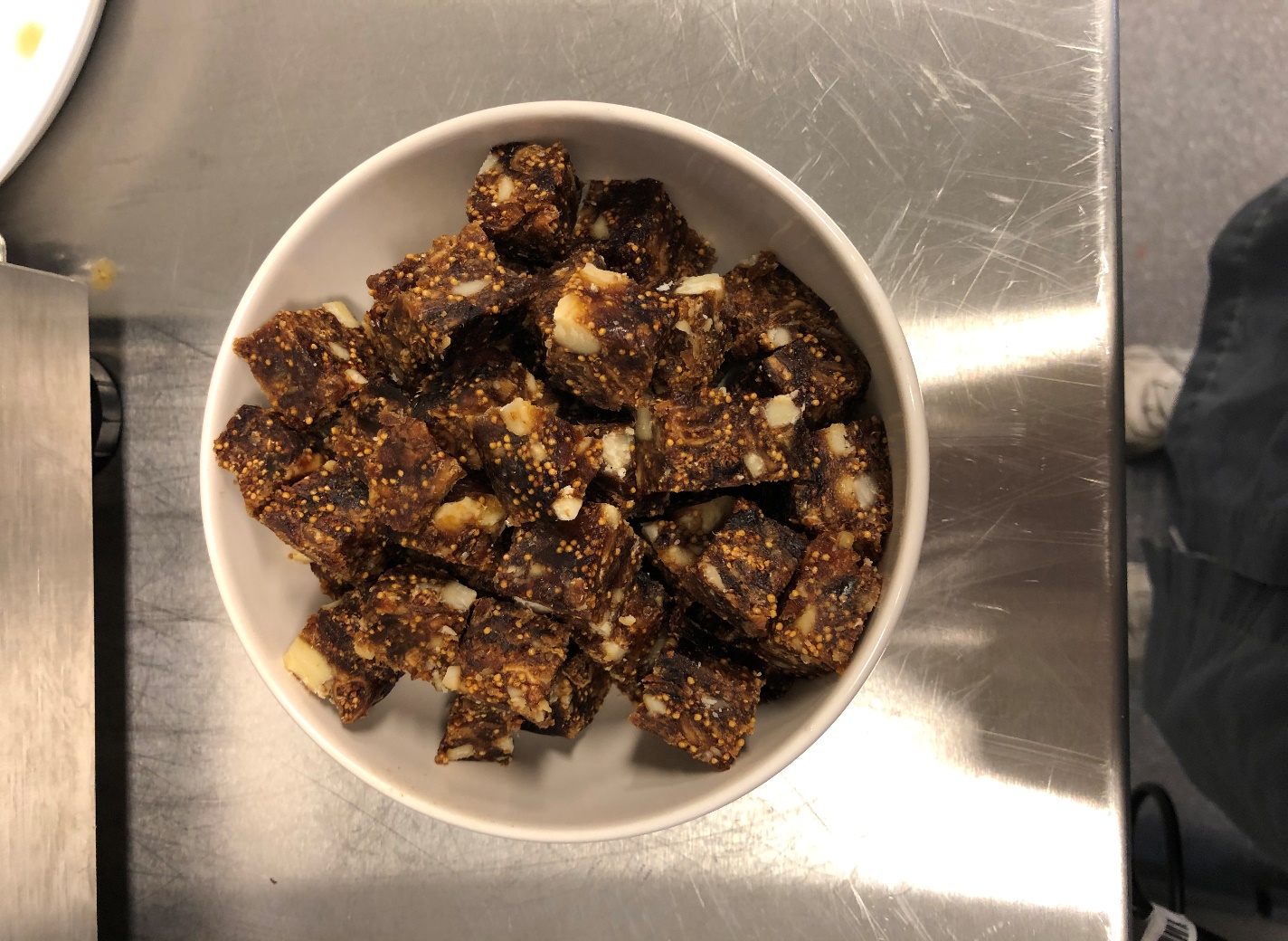
**(ultra-)processed, Soft texture- Menu**

**Breakfast**

| Ingredients | Weight% of total weight (1880 gram) | Kcal%  of total (810 kcal) | NOVA class | Number of ingredients incl. additives | Product type or brand |
| --- | --- | --- | --- | --- | --- |
| Fruit smoothie | 71.8 | 100.0 | 3 | 16 | Innocent super smoothie energise |
| Water served at the side | 28.2 | 0.0 | 1 | - | tap water |


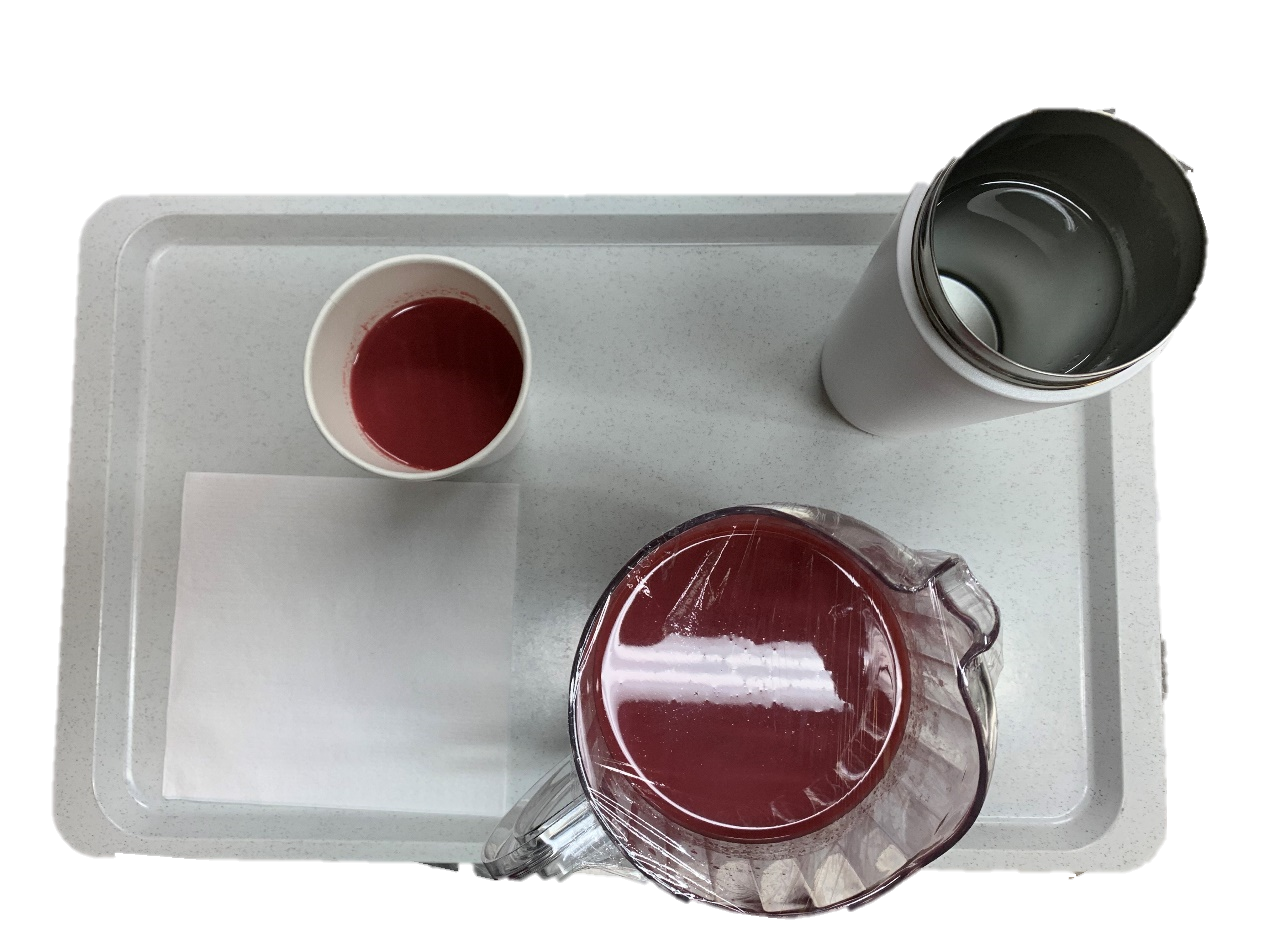


**Morning Snack**

| Ingredients | Weight% of total weight (90 gram) | Kcal%  of total (41 kcal) | NOVA class | Number of ingredients incl. additives | Product type or brand |
| --- | --- | --- | --- | --- | --- |
| apple juice from concentrate | 100 | 100 | 4 | 1 | Apple, from concentrate, Jumbo |

**Lunch**

| Ingredients | Weight% of total weight (1100 gram) | Kcal%  of total (1567 kcal) | NOVA class | Number of ingredients incl. additives | Product type or brand |
| --- | --- | --- | --- | --- | --- |
| Cheese Goudse belegen 48+ | 90.9 | 75.9 | 3 | 5 | Goudse mature cheese 48+ |
| ready-to-eat macaroni bolognese | 9.1 | 24.1 | 4 | 31 | Macaroni bolognese Jumbo |


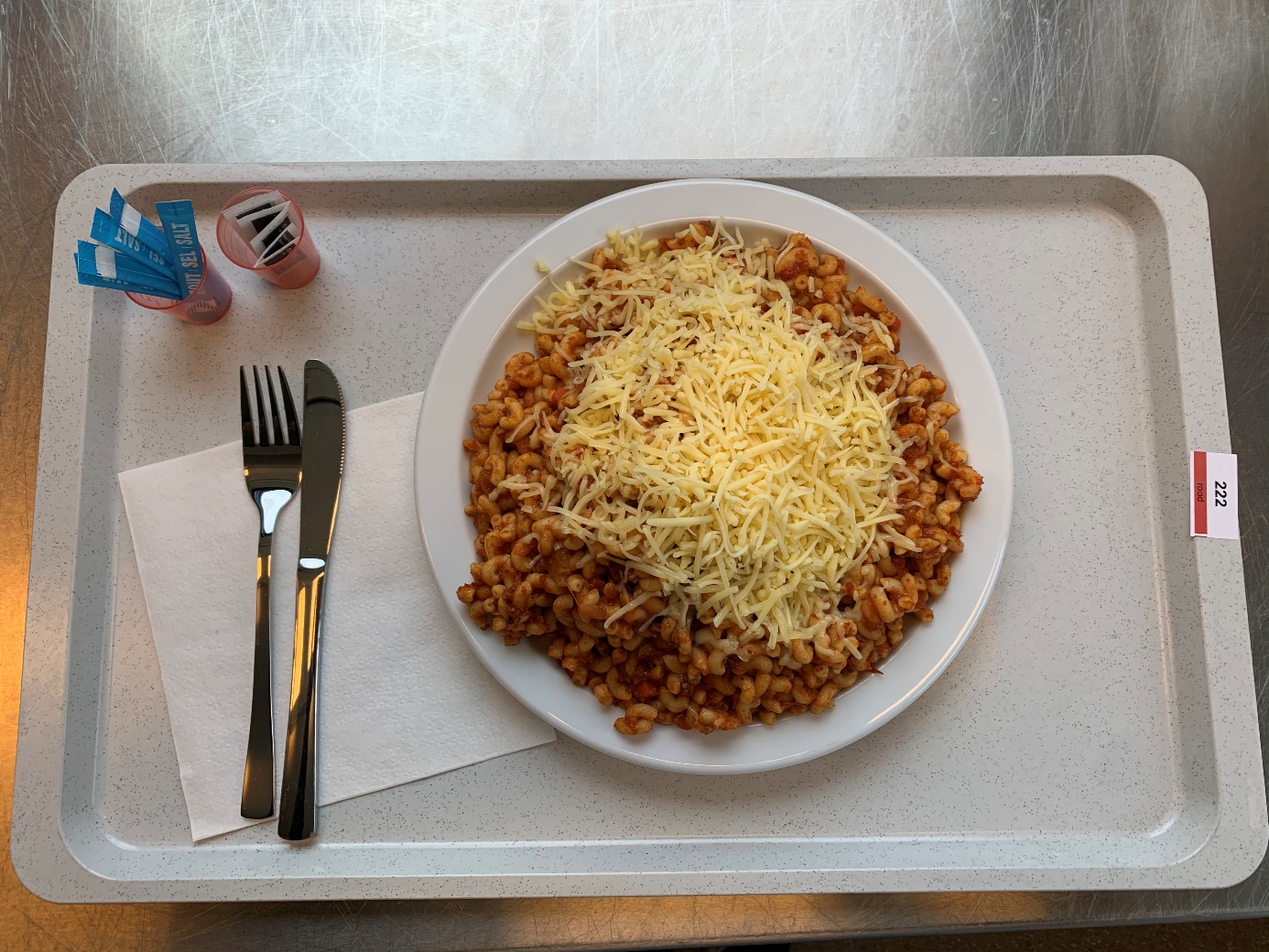


**Afternoon Snack**

| Ingredients | Weight% of total weight (50 gram) | Kcal%  of total (195 kcal) | NOVA class | Number of ingredients incl. additives | Product type or brand |
| --- | --- | --- | --- | --- | --- |
| Fruit bar | 100 | 100 | 4 | 29 | Albert Heijn Fruit reep(bar) appel |

**Dinner**

| Ingredients | Weight% of total weight (845 gram) | Kcal%  of total (733 kcal) | NOVA class | Number of ingredients incl. additives | Product type or brand |
| --- | --- | --- | --- | --- | --- |
| green beans canned cooked | 35.5 | 10.2 | 3 | 3 | HAK green beans pieces |
| ready-to-eat potato puree | 35.5 | 29.9 | 4 | 8 | Potato puree a la minute, creme fraiche with chives, Maggi |
| chicken meat balls prepared | 17.2 | 36.6 | 4 | 14 | Albert Heijn  Chicken meatbals |
| creme fraiche | 5.9 | 20.1 | 1 | 1 | Albert Heijn, Fresh creme fraiche |
| AH sajoer | 5.9 | 3.3 | 4 | 37 | AH boemboe sajoer beans |


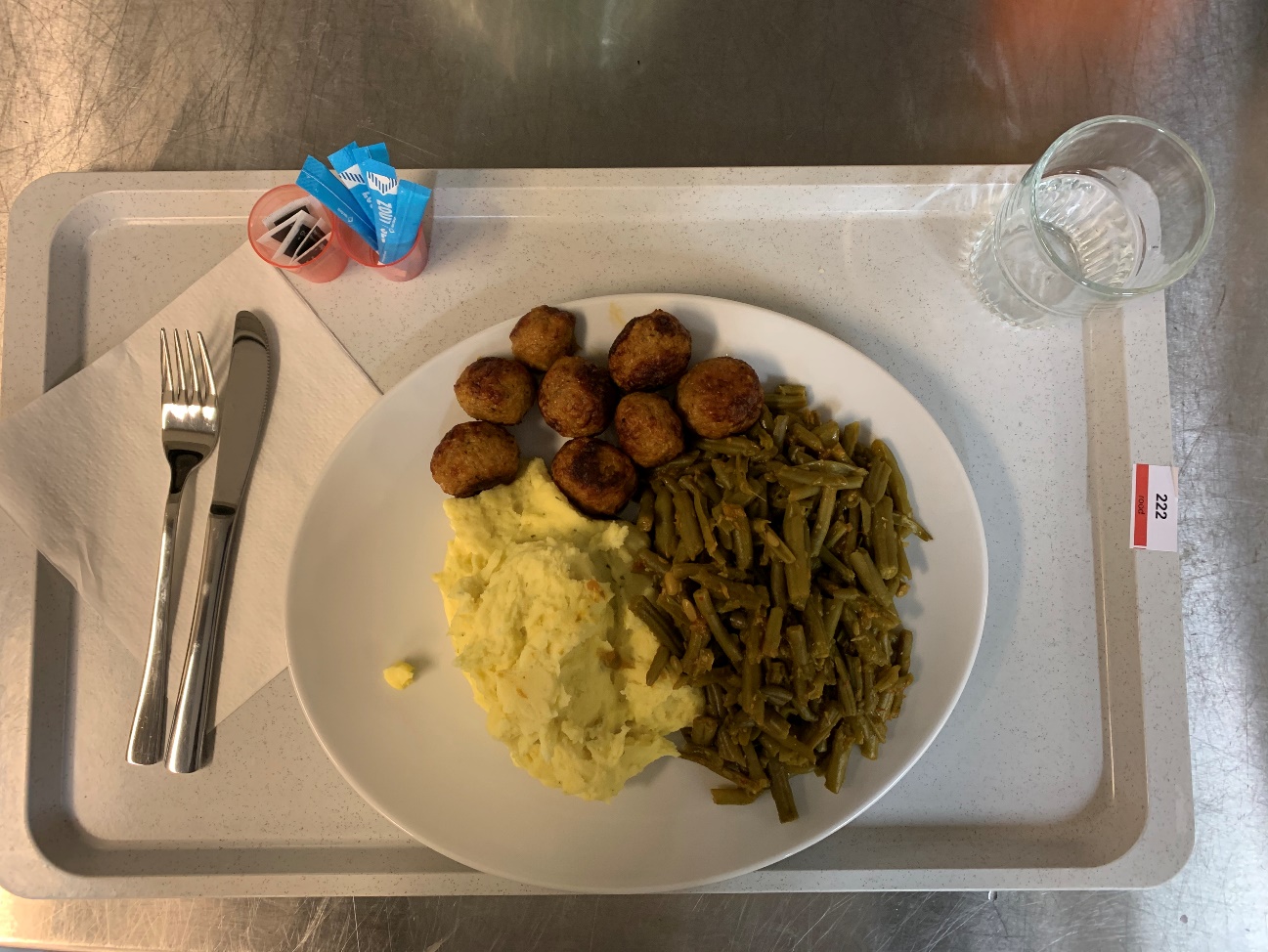
**Dessert**

| Ingredients | Weight% of total weight (350 gram) | Kcal%  of total (1089 kcal) | NOVA class | Number of ingredients incl. additives | Product type or brand |
| --- | --- | --- | --- | --- | --- |
| yoghurt walnut-honey flavour | 71.4 | 33.8 | 4 | 7 | Room yoghurt walnut and greek honey, Almhof |
| pecan nuts unsalted | 28.6 | 66.2 | 1 | 1 | - |


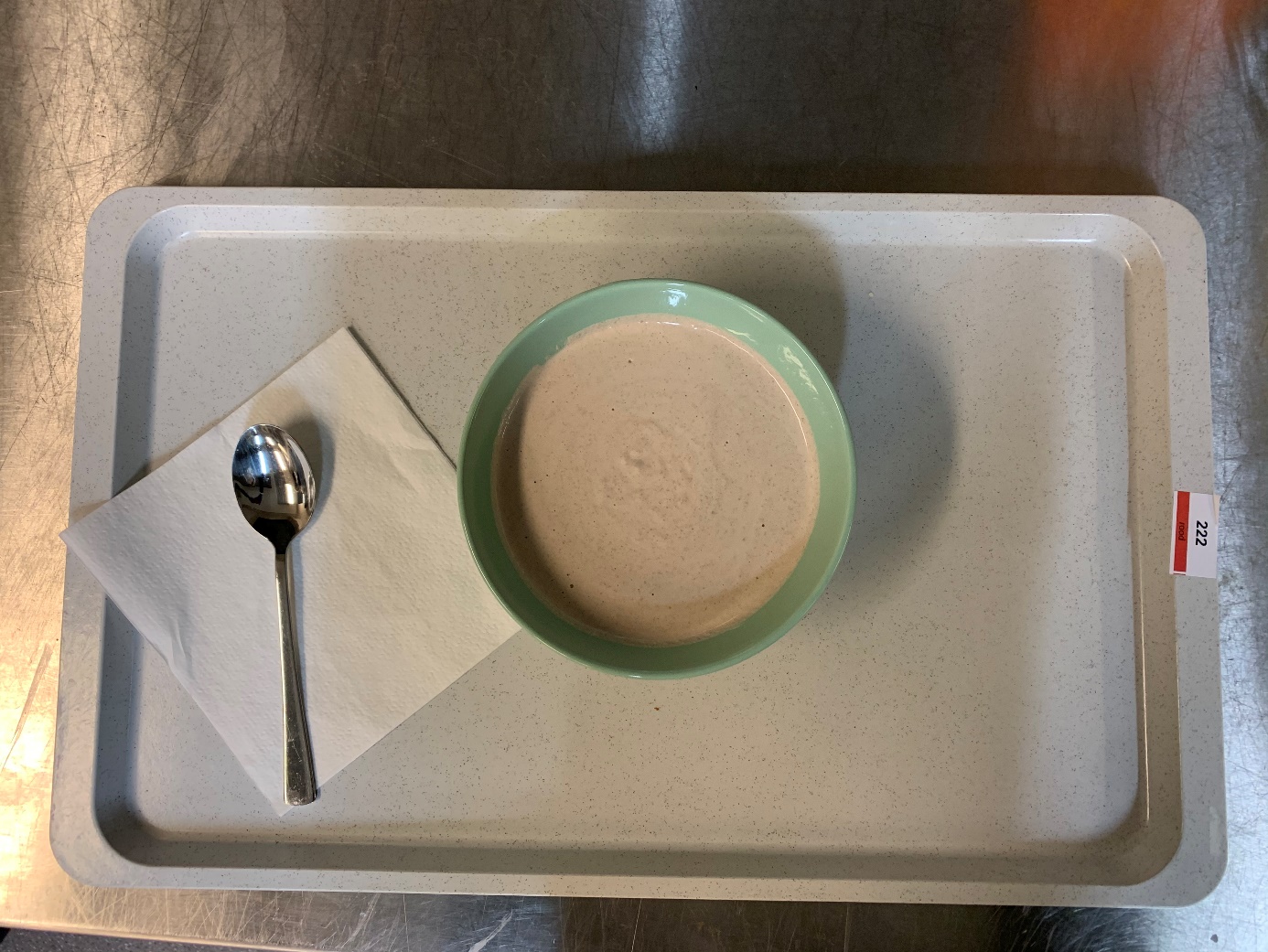


**Supplement 2- Diet composition of the study meals per condition**

Table 1. Diet composition of the study meals (as offered) of each of the conditions, per main meal and snack.

| Meal |  | Unprocessed | | Processed | |
| --- | --- | --- | --- | --- | --- |
|  |  | Hard | Soft | Hard | Soft |
| Breakfast | Weight (g) | 1888 | 1888 | 1880 | 1880 |
|  | Energy kJ(kcal) | 3721(882) | 3721(882) | 3463(820) | 3402(810) |
|  | Energy density (kcal/g) | 0.47 | 0.47 | 0.44 | 0.43 |
|  | Energy density (kcal/g) *excl. water on the side | 0.70 | 0.47 | 0.61 | 0.60 |
|  | Fat (g (en%)) | 5.3(5.3) | 5.3(5.3) | 0 | 12.2(13.2) |
|  | Saturated Fat (g) | 0.7 | 0.7 | 0 | 1.4 |
|  | Carbohydrate (g(en%)) | 185.1(84.6) | 185.1(84.6) | 189(92.8) | 162(81) |
|  | Mono- disaccharides (g) | 158.5 | 158.5 | 149 | 135 |
|  | Protein (g(en%)) | 8.6(3.9%) | 8.6(3.9%) | 3.8(1.8%) | 8.1(4.0%) |
|  | Fiber (g) | 26 | 26 | 10.2 | 6.8 |
|  | Sodium (mg) | 30 | 30 | 12 | 11 |
| Morning snack | Weight (g) | 90 | 90 | 90 | 90 |
|  | Energy kJ(kcal) | 209(50) | 182(43) | 209(50) | 175(41) |
|  | Food energy density (kcal/g) | 0.56 | 0.48 | 0.56 | 0.46 |
|  | Fat (g (en%)) | 0.2(3.2) | 0 | 0.2(3.2) | 0 |
|  | Saturated Fat (g) | - | - | - | - |
|  | Carbohydrate (g(en%)) | 10.8(87.9) | 9.6(90) | 10.8(87.9) | 10.1(98.1) |
|  | Mono- disaccharides (g) | 9.2 | 8.9 | 9.2 | 9.5 |
|  | Protein (g(en%)) | 0.3(2.2) | 0.2(1.7) | 0.3(2.2) | 0.1(0.9) |
|  | Fiber (g) | 1.7 | 1.3 | 1.7 | 0 |
|  | Sodium (mg) | 1 | 1 | 1 | 4 |
| Lunch | Weight (g) | 1078 | 1078 | 1260 | 1100 |
|  | Energy kJ (kcal) | 6715(1608) | 6715(1608) | 6695(1597) | 6555(1567) |
|  | Food energy density (kcal/g) | 1.49 | 1.49 | 1.27 | 1.42 |
|  | Fat (g(en%)) | 83(45.9) | 83(45.9) | 77(42.5) | 68(38.3) |
|  | Saturated Fat (g) | 15.9 | 15.9 | 42.4 | 32 |
|  | Carbohydrate (g(en%)) | 115.2(29.2) | 115.2(29.2) | 133.4(33.9) | 162(42) |
|  | Mono- disaccharides (g) | 55.3 | 55.3 | 32 | 25 |
|  | Protein (g(en%)) | 91.5 (23.2) | 91.5 (23.2) | 79.6(20.2) | 68.5(17.8) |
|  | Fiber (g) | 14.6 | 14.6 | 16.1 | 11 |
|  | Sodium (mg) | 238 | 238 | 3725 | 3818 |
| Afternoon snack | Weight (g) | 50 | 50 | 50 | 50 |
|  | Energy kJ(kcal) | 723(170) | 689(163) | 818(195) | 820(195) |
|  | Food energy density (kcal/g) | 3.4 | 3.26 | 3.90 | 3.90 |
|  | Fat (gram(en%)) | 0.5(2.3) | 0.3(1.3) | 6.5(29.4) | 4.3(19.2) |
|  | Saturated Fat (g) | 0 | 0.1 | 1.3 | 2 |
|  | Carbohydrate (g(en%)) | 39.5(92.9) | 35.9(88.5) | 24(49.9) | 36(74.6) |
|  | Mono- disaccharides (g) | 32.5 | 34.5 | 5.5 | 23 |
|  | Protein (g(en%)) | 1(2.4) | 1.6(3.8) | 4(8.3) | 2.3(4.7) |
|  | Fiber (g) | 2.3 | 1.9 | 12.5 | 1.5 |
|  | Sodium (mg) | 0 | 13 | 20 | 60 |

| Meal |  | Unprocessed | | Processed | |
| --- | --- | --- | --- | --- | --- |
|  |  | Hard | Soft | Hard | Soft |
| Dinner | Weight (g) | 831 | 831 | 820 | 845 |
|  | Energy kJ (kcal) | 2880(685) | 3069(731) | 3221(769) | 3072(733) |
|  | Food energy density (kcal/g) | 0.82 | 0.88 | 0.94 | 0.87 |
|  | Fat (g (en%)) | 11.9(15.3) | 30.4(36.7) | 25(28.7) | 38(45.8) |
|  | Saturated Fat (g) | 3.7 | 14.7 | 5 | 18.2 |
|  | Carbohydrate (g(en%)) | 69.4(41) | 75.7(41.9) | 95.5(50.4) | 55.9(30.9) |
|  | Mono- disaccharides (g) | 23 | 21.5 | 11 | 10.6 |
|  | Protein (g(en%)) | 67.6(39.9) | 31.7(17.6) | 32.6(17.2) | 35.1(19.4) |
|  | Fiber (g) | 14.5 | 14.3 | 15.4 | 14.5 |
|  | Sodium (mg) | 170 | 275 | 1882 | 789 |
| Dessert | Weight (g) | 340 | 350 | 340 | 350 |
|  | Energy kJ (kcal) | 4410(1042) | 4306(1040) | 4502(1074) | 4504(1089) |
|  | Food energy density (kcal/g) | 3.06 | 2.97 | 3.16 | 3.11 |
|  | Fat (g(en%)) | 18.8(15.7) | 84.6(72.7) | 27.9(22.9) | 94(77.2) |
|  | Saturated Fat (g) | 1.9 | 14.6 | 2 | 20 |
|  | Carbohydrate (g(en%)) | 185(71.3) | 45(17.8) | 149.6(56.5) | 37.4(14.1) |
|  | Mono- disaccharides (g) | 171.9 | 44.6 | 128.9 | 34.5 |
|  | Protein (g (en%)) | 15.6(6.0) | 19.9(7.8) | 28.9(10.9) | 16.5(6.2) |
|  | Fiber (g) | 37.3 | 9.6 | 54.4 | 9.6 |
|  | Sodium (mg) | 1 | 84 | 544 | 0 |

**Supplement 3- Food intake and eating behaviour per meal and study condition**

Table 1. Food intake, energy intake and eating behaviour characteristics-per main meal

|  | Unprocessed | | (Ultra-)processed | | **ANOVA Fixed Effect *P* - value** | | |
| --- | --- | --- | --- | --- | --- | --- | --- |
|  | Hard | Soft | Hard | Soft | Proce-ssing | Texture | Processing* Texture |
| Breakfast | | | | | | | |
| Food intake (kcal) | 343±41^a^ | 316±41^a^ | 261±41^a^ | 424±42^b^ | 0.58 | 0.01 | 0.001 |
| Food intake (g) | 458±71 | 591±71 | 397±71 | 586±72 | 0.34 | <0.001 | 0.40 |
| Water intake (g) | 245±29^a^ | No water offered | 192±29^a,b^ | 98±29^b^ | 0.37 | <0.001 | 0.007 |
| Energy intake rate (kcal/min) | 29±4^a^ | 34±4^a^ | 27±4^a^ | 55±4^b^ | 0.003 | 0.001 | 0.002 |
| Eating rate (g/min) | 41±7 | 72±7 | 45±7 | 92±7 | 0.03 | <0.001 | 0.11 |
| Total meal duration (min) | 13±1 | 10±1 | 10±1 | 8±1 | <0.001 | <0.001 | 0.32 |
| nOSE duration (s/bite) | 18±2 | 10±2 | 17±2 | 8±2 | 0.16 | <0.001 | 0.26 |
| Chews per bite | 18±2 | 3±2 | 19±2 | 1±2 | 0.83 | <0.001 | 0.48 |
| Bite size (g) | 14±3 | 24±3 | 16±3 | 33±3 | 0.03 | <0.001 | 0.14 |
| Lunch | | | | | | | |
| Food intake (kcal) | 1106±97 | 959±97 | 820±100 | 1074±100 | 0.36 | 0.56 | 0.04* |
| Food intake (g) | 699±48 | 595±48 | 635±48 | 605±48 | 0.42 | 0.04* | 0.25 |
| Water intake (g) | 285±38 | 306±38 | 294±38 | 302±38 | 0.91 | 0.54 | 0.79 |
| Energy intake rate (kcal/min) | 57±8 | 73±8 | 60±8 | 88±8 | 0.15 | 0.002 | 0.31 |
| Eating rate (g/min) | 39±5 | 49±5 | 47±5 | 54±5 | 0.01 | 0.002 | 0.56 |
| Total meal duration (min) | 19±1 | 14±1 | 16±1 | 13±1 | <0.001 | <0.001 | 0.07 |
| OSE duration (sec/bite) | 16±2 | 13±2 | 13±2 | 13±2 | 0.02* | 0.15 | 0.13 |
| Chews per bite | 16±3 | 16±3 | 13±3 | 15±3 | 0.41 | 0.78 | 0.52 |
| Bite size (g) | 14±1 | 16±1 | 13±1 | 15±1 | 0.09 | 0.05 | 0.81 |
| Sodium in meal (mg) | 148±129 | 128±117 | 1994±125 | 2246±129 | <0.001 | 0.29 | 0.23 |
| Sodium added (mg) | 203±47 | 260±43 | 10±46 | 97±47 | <0.001 | 0.06 | 0.69 |
| Sodium total intake (mg) | 350±139 | 389±128 | 2001±136 | 2342±139 | <0.001 | 0.09 | 0.18 |

|  | Unprocessed | | | | (Ultra-)processed | | | **ANOVA Fixed Effect *P* - value** | | |  |
| --- | --- | --- | --- | --- | --- | --- | --- | --- | --- | --- | --- |
|  | Hard | | Soft | Hard | | Soft | | processing | Texture | Processing* Texture |  |
| Dinner | | | | | | | | | | | |
| Food intake (kcal) | 313±34 | | 391±34 | 323±34 | | 513±34 | | 0.02 | <0.001 | 0.05 |  |
| Food intake total (g) | 386±34 | | 496±35 | 444±35 | | 563±35 | | 0.01 * | <0.001 | 0.89 |  |
| Beans | 173±19 | | 174±20 | 161±20^a^ | | 228±20^b^ | | 0.17 | 0.03 | 0.04 |  |
| Potato | 143±18 | | 216±18 | 155±18 | | 228±18 | | 0.44 | <0.001 | 0.997 |  |
| Protein  (meat/egg) | 82±9^a^ | | 121±9^b^ | 145±9^b^ | | 131±9^b^ | | <0.001 | 0.10 | 0.003 |  |
|  |  | |  |  | |  | |  |  |  |  |
| Water intake (g) (dinner and dessert) | 423±52 | | 328±52 | 517±52 | | 341±52 | | 0.16 | <0.001 | 0.29 |  |
| Energy intake rate (kcal/min) | 17±3 | | 38±3 | 18±3 | | 46±3 | | 0.07 | <0.001 | 0.20 |  |
| Eating rate (g/min) | 22±3 | | 47±3 | 26±3 | | 50±3 | | 0.18 | <0.001 | 0.88 |  |
| Total meal duration (min) | 18±1 | | 12±1 | 18±1 | | 13±1 | | 0.45 | <0.001 | 0.50 |  |
| OSE duration (s/bite) | 16±2 | | 11±2 | 15±2 | | 12±2 | | 0.97 | <0.001 | 0.31 |  |
| Chews per bite | 20±3 | | 11±3 | 17±3 | | 12±3 | | 0.59 | <0.001 | 0.20 |  |
| Bite size (g) | 7±1 | | 12±1 | 9±1 | | 14±1 | | 0.004 | <0.001 | 0.22 |  |
| Sodium in meal (mg) | 36±17^a,c^ | | 77±18^a^ | 517±18^b^ | | 447±18^b,d^ | | <0.001 | 0.38 | 0.003 |  |
| Sodium added (mg) | 106±40 | | 173±41 | 100±41 | | 111±41 | | 0.24 | 0.18 | 0.34 |  |
| Sodium total intake (mg) | 141±48 ^a,c^ | | 249±48 ^a^ | 615±48^b^ | | 558±48 ^b,d^ | | <0.001 | 0.41 | 0.02 |  |
| Dessert | | | | | | | | | | | |
| Food intake (kcal) | | 195±40 | 444±41 | | 162±41 | | 501±41 | 0.77 | <0.001 | 0.28 |  |
| Food intake (g) | | 50±13 | 134±13 | | 40±13 | | 144±13 | 0.64 | <0.001 | 0.38 |  |
| Energy intake rate (kcal/min) | | 62±18 | 118±18 | | 50±18 | | 147±18 | 0.45 | <0.001 | 0.09 |  |
| Eating rate (g/min) | | 18±6 | 39±7 | | 15±6 | | 54±6 | 0.23 | <0.001 | 0.09 |  |
| Total meal duration (min) | | 5±1 | 5±1 | | 4±1 | | 4±1 | 0.10 | 0.49 | 0.74 |  |
| OSE duration (sec/bite) | | 16±2 | 11±2 | | 15±2 | | 12±2 | 0.97 | <0.001 | 0.32 |  |
| Chews per bite | | 25±3 | 6±3 | | 35±3 | | 5±3 | 0.59 | <0.001 | 0.20 |  |
| Bite size (g) | | 8 | 11 | | 9 | | 11 | 0.84 | 0.06 | 0.58 |  |

*no differences between conditions (based on post hoc test), OSE= oro-sensory exposure
